# Murine Model for Measuring Effects of Humanized-Dosing of Antibiotics on the Gut Microbiome

**DOI:** 10.3389/fmicb.2022.813849

**Published:** 2022-02-17

**Authors:** Shana R. Leopold, Kamilia Abdelraouf, David P. Nicolau, Hanako Agresta, Jethro Johnson, Kathleen Teter, Wm Michael Dunne, David Broadwell, Alex van Belkum, Lisa M. Schechter, Erica J. Sodergren, George M. Weinstock

**Affiliations:** ^1^The Jackson Laboratory for Genomic Medicine, Farmington, CT, United States; ^2^Center for Anti-Infective Research and Development, Hartford Hospital, Hartford, CT, United States; ^3^BioMérieux Inc., Durham, NC, United States; ^4^BioMérieux SA, Clinical Unit, Grenoble, France; ^5^BioMérieux SA, Clinical Unit, Hazelwood, MO, United States

**Keywords:** humanized antibiotic treatment, piperacillin-tazobactam, non-infected mouse model, microbiome dynamics, metabolome, gastro-intestinal tract

## Abstract

There is a current need for enhancing our insight in the effects of antimicrobial treatment on the composition of human microbiota. Also, the spontaneous restoration of the microbiota after antimicrobial treatment requires better understanding. This is best addressed in well-defined animal models. We here present a model in which immune-competent or neutropenic mice were administered piperacillin-tazobactam (TZP) according to human treatment schedules. Before, during and after the TZP treatment, fecal specimens were longitudinally collected at established intervals over several weeks. Gut microbial taxonomic distribution and abundance were assessed through culture and molecular means during all periods. Non-targeted metabolomics analyses of stool samples using Quadrupole Time of Flight mass spectrometry (QTOF MS) were also applied to determine if a metabolic fingerprint correlated with antibiotic use, immune status, and microbial abundance. TZP treatment led to a 5–10-fold decrease in bacterial fecal viability counts which were not fully restored during post-antibiotic follow up. Two distinct, relatively uniform and reproducible restoration scenarios of microbiota changes were seen in post TZP-treatment mice. Post-antibiotic flora could consist of predominantly Firmicutes or, alternatively, a more diverse mix of taxa. In general, the pre-treatment microbial communities were not fully restored within the screening periods applied. A new species, closely related to *Eubacterium siraeum*, *Mageeibacillus indolicus*, and *Saccharofermentans acetigenes*, became predominant post-treatment in a significant proportion of mice, identified by 16S rRNA gene sequencing. Principal component analysis of QTOF MS of mouse feces successfully distinguished treated from non-treated mice as well as immunocompetent from neutropenic mice. We observe dynamic but distinct and reproducible responses in the mouse gut microbiota during and after TZP treatment and propose the current murine model as a useful tool for defining the more general post-antibiotic effects in the gastro-intestinal ecosystem where humanized antibiotic dosing may ultimately facilitate extrapolation to humans.

## Introduction

Antimicrobial agents are crucial for protection against and cure of infectious processes. Many different categories of antibiotics have been developed to date and most have found limited or prolonged success. Currently, overall antibiotic effectivity seems to be waning due to the global development of antimicrobial resistance (AMR) and a reduced developmental pipeline for new antibiotics (D’Atri et al., 2019). This trend is worrisome and one that needs vigorous attention. There is a need for alternative approaches next to the purely (bio-)chemical ones that have dominated. The use of bacteriolytic enzymes, clever nucleic acid constructs, or bacteriophages for combatting infections are currently being examined as alternative approaches that target specific pathogenic species and spare colonizing bacterial populations present in human microbiota and not involved in the actual disease process (Gordillo Altamirano and Barr, 2019; López-Jácome et al., 2019). In the meantime, the effect of antibiotic treatment on mixed bacterial populations needs further clarification (Galera-Laporta and Garcia-Ojalvo, 2020).

Recently, our knowledge on the nature of the human microbiomes has been systematically improved and its overall relevance to health is now an accepted paradigm (Schloissnig et al., 2013; Sunagawa et al., 2014). A healthy and diverse gastro-intestinal bacterial flora, for instance, has been suggested to provide a natural barrier against infection (Sassone-Corsi and Raffatellu, 2015). Hence, fecal transplantation is an alternative means to curing certain infections by repairing the stasis of the patient’s microbiome. Still, the Yin-Yang effects of antibiotic use vs. targeted microbiome interventions, or their combination is an emerging science and detailed mechanistic and therapeutic studies have yet to be completed.

In addition to targeting pathogens, antibiotics also alter the resident commensal intestinal microbiota, often resulting in a dysbiotic gut microbiome. These effects are host dependent and may have differing influences on individual metabolic processes (Fujisaka et al., 2016). Antibiotic treatment may help selectively amplify certain sub-populations of potentially pathogenic bacterial species and/or enrich antibiotic resistance genes within the population (Ubeda et al., 2010; Meletiadis et al., 2017; Richter et al., 2018; Burdet et al., 2019; Haak et al., 2019). Antibiotics can promote disease by themselves (antibiotic-associated diarrhea, for instance; Rashid et al., 2015) and protection from those unwanted side effects are important, possibly through microbiome-mediated interventions (Becattini et al., 2017; De Gunzburg et al., 2018). Overall, antimicrobial agents can potentially affect the stability and complexity of microbiomes in both the short- and long-term. More detailed studies are required to define whether antibiotic-induced disturbance of microbiomes can be normalized once therapy has been discontinued. So called dysbiome or dysbiosis studies have revealed novel associated pathologies (Carding et al., 2015; Gilbert et al., 2018; Janssens et al., 2018). Studying the effects of antibiotics on the microbiome is complicated in the very diverse human ecosystem, including the added variations of human diet, host genetics, prior microbiome perturbations, and variations in the microbiome composition from one person to the next.

Here, we advocate an approach that uses a more standardized longitudinal murine model to study the effect of humanized-dosing of piperacillin-tazobactam (TZP) on the gut microbiome in a controlled physiological environment (Bulik and Nicolau, 2010; Bulik et al., 2012). We used TZP because its pharmacokinetic and pharmacodynamic (PK/PD) characteristics in humans are well-known. In addition, TZP is a frequently used beta-lactam/beta-lactamase inhibitor combination. The use of dosing schemes meant to mimic the PK/PD of antibiotics in humans improves the translational value of the murine model since historic approaches use drug exposures that are not achieved in humans and, therefore, not clinically useful (Gill et al., 2020). We performed a taxonomic survey of the mouse fecal microbiome using 16S rRNA gene sequencing before, during and after TZP treatment. This study showed the bacterial taxa present in terms of percentages as well as the quantitative bacterial load, which was more precisely defined using quantitative PCR (qPCR). In addition, we performed metabolomic studies and defined the parallel between microbiological variation and the (im)balance within the metabolome.

## Materials and Methods

### Immune-Competent Uninfected Animal Model

Two experiments were conducted in which TZP was administered to mice in a “humanized” dosing regimen. The first pilot experiment (TZP1) consisted of 6 days pre-TZP monitoring (including 48 h of acclimation after arrival to the vivarium from the vendor), 7 days of TZP treatment, and 7 days post-TZP treatment follow-up. After conducting preliminary analyses on the fecal pellets from this experiment, we designed a second experiment (TZP2) in which two sets of mice, immunocompetent and neutropenic, were allowed a 14-day pre-TZP time period, to observe bacterial community changes during the acclimation period, followed by 4 days of TZP exposure using the humanized dosing regimen, and 21 days of post-antibiotic recovery period.

For each experiment, 10 specific-pathogen-free, female ICR (CD-1) mice weighing between 20 and 22 g upon delivery were obtained from Envigo RMS, Inc. (Indianapolis, Indiana). In the second experiment, 10 neutropenic mice were included as well (see below in section “Neutropenic Uninfected Animal Model”). Upon receipt from the vendor, animals were housed individually to prevent the exchange of genetic material through the consumption of fecal droppings. All animals were provided food and water *ad libitum*. After the 48-h acclimation period, fecal pellets were collected from each mouse over a period of 2 h (Day 2) during which each mouse was individually housed in a box without bedding, food, or water. After fecal pellet collection, mice were placed into new individual cages. Pellets for culture (first experiment only) were placed into Copan liquid Amies Elution tubes (Copan Italia, Brescia, Italy) for collection and transport and stored at 4°C–8°C until transport to the Jackson Laboratory, where they were again stored at 4°C until culture. Pellets for microbiome analysis were stored at −80°C until transport to the Jackson Laboratory on dry ice, where they were then stored at −80°C. Additionally, pellets were collected and stored at −80°C to be assayed for TZP concentrations. Fecal collection was repeated at the end of days 6, 9, 13, and 20 for TZP1 and daily (days 0–39) for TZP2. Animals were euthanized using CO_2_ and cervical dislocation at the end of the study period.

### Neutropenic Uninfected Animal Model

TZP2 included 10 immunocompetent and 10 neutropenic mice. For the neutropenic model, 10 mice were housed individually, and fecal samples were collected daily as described above for the immunocompetent uninfected animal model, except that the mice were rendered transiently neutropenic by injecting cyclophosphamide intra-peritoneally (IP) at a dose of 250 mg/kg of body weight at 4 days and 100 mg/kg 1 day before antibiotic dosing began. Transient neutropenia lasts for about 10–11 days as based on long-lasting experience at Jackson Laboratories.

### Human-Simulated Dosing Regimen

Commercially available TZP was obtained from Cardinal Health (Dublin, OH). Dosing solutions were prepared in sterile 0.9% normal saline. All dosing solutions were held on ice, protected from light throughout the dosing period. The mice received the following TZP dosing regimen subcutaneously to achieve exposures comparable to those achieved in humans: 500/62.5 mg/kg at 0 h, 100/12.5 mg/kg at 0.25 h, 200/25 mg/kg at 2.5 h, and 75/9.375 mg/kg at 5 h (Bulik et al., 2012). This dosing regimen was repeated every 6 h for the duration of the antibiotic exposure portion of the experiment (TZP1: Days 7–13, TZP2: Days 15–18).

### Determination of Piperacillin Content in Murine Feces

Piperacillin concentrations were determined using a validated high-performance liquid chromatography (HPLC) assay, modified from a previously reported method (Kim et al., 2002), at the Center for Anti-Infective Research and Development (CAIRD), Hartford Hospital. The mobile phase consisted of 0.1 M phosphate buffer pH 2.7 with 29.8% acetonitrile (ACN). The standards and quality control samples were prepared in pooled mouse feces matrix. Mouse feces samples were weighed and a mixture of methanol, water, and acetic acid (40:40:1) was added to achieve a 1:8 dilution. An internal standard of 20 μl of 2,500 μg/ml penicillin G (Sigma, St. Louis, MO) and stainless steel 3.2 mm beads (Biospec Products Bartlesville, United Kingdom) were added, the sample was vortexed then centrifuged, and the supernatant was assayed for piperacillin content. The HPLC system consisting of a Waters 515 pump (Waters Associates, Milford, MA, United States) and a 717 plus auto-sampler (Waters) was equipped with a 10 μm Prodigy C18 column (4.6 mm × 250 mm, Phemonenex Inc., Torrance, CA, United States) coupled to a Bondapak C_18_ Guard-pak pre-column (Waters). A programmable UV detector (Model 526; ESA Inc., Chelmsford, MA) set at 230 nm for 23 min was used to detect the analytes. The lower limit of quantification was 16 μg/g of feces. Optimized intraday coefficients of variation for the low and high quality control samples (*n* = 10) were 3.2 and 2.4%, respectively. Inter-day coefficients of variation were 6.0 and 5.0%, respectively (*n* = 7).

### 16S Microbiome Sequencing and Operational Taxonomic Unit Generation

DNA was extracted from stool specimens using the MoBio PowerSoil DNA Isolation Kit (Carlsbad, United States). Targeted amplification of the V1–V3 hypervariable region of the 16S rRNA gene was performed with primers 27F (5′-AGAGTTTGATCCTGGCTCAG-3′) and 534R (5′-ATTACCGCGGCTGCTGG-3′) using AccuPrime Taq DNA Polymerase High Fidelity (Invitrogen, Waltham, United States) and 4 ng of template DNA in a total reaction volume of 20 μl. The PCR was run using the following cycling parameters: 2 min of denaturation at 95°C, followed by 30 cycles of 20 s at 95°C, 30 s at 56°C, and 1 min at 72°C. The presence of amplicons was confirmed by agarose gel electrophoresis and staining with GreenGlo Safe DNA Dye (Thomas Scientific, Santa Clare, CA, United States). The concentrations of amplicons were quantified by Qubit dsDNA HS Kit (Invitrogen), and then equimolar amounts (4 nM) of the PCR amplicons were pooled in a single tube. Short DNA fragments and amplification primers were removed from the pooled amplicons using AMPure XP beads (Beckman-Coulter, Indianapolis, IN, United States), and subsequently sequenced using 2 × 300 base-pair paired-end sequencing (Illumina MiSeq; Illumina, San Diego, United States). Proprietary Illumina software was applied to perform initial processing of raw sequencing data, removing barcodes and primers. A standard of one mismatch in primer and zero mismatch in barcode was applied to assign read pairs to the appropriate sample in a pool of samples. Adapters and low-quality bases were trimmed using Trimmomatic (Bolger et al., 2014), paired end sequences were assembled with FLASH (Magoč and Salzberg, 2011), and chimeras were removed with UCHIME (Edgar et al., 2011). Mouse contaminant sequences were filtered out using BMTagger (Lee and Shatkay, 2006). The resulting sequences were clustered into 97% similarity operational taxonomic units (OTUs) using USEARCH v10.0.240 (Wang et al., 2007; Edgar et al., 2011), and final taxonomic assignment was performed using the RDP Classifier (Wang et al., 2007). OTUs containing mitochondrial sequences were identified by BLAST (Camacho et al., 2009) against a local database of bacterial and mitochondrial RefSeq 16S sequences downloaded from NCBI GenBank on January 25, 2019. OTUs where the reference sequence had >97% identity and >97% query coverage to a mitochondrial sequence were removed from further analysis.

In several cases, the partial V1–V3 16S rRNA gene sequence indicated that a single bacterial species was predominant in the specimen. We directly sequenced the entire V1–V9 16S rRNA gene (Johnson et al., 2019; primers 27F, AGAGTTTGATCMTGGCTCAG, and 1492R, TACGGYTACCTTGTTAYGACTT) from such stools at the antibiotic treatment time point for three mice: mouse 6 in TZP1, and mice 9 (immunocompetent) and 19 (neutropenic) from TZP2. The high-quality reads from these three samples were pooled and OTUs were formed. The 10 OTUs with the most reads assigned were then matched against the GenBank RefSeq collection using BLAST to find the closest hits (the nearest match was ~84%). Those closest hits were used to build a phylogenetic tree using the neighbor-joining method.

### Bacterial Culturing

To identify any low- or high-level TZP resistant aerobic organisms, liquid from each Copan transport tube was plated onto CHROMID agar (bioMérieux, Durham, NC, United States) with 4/4 μg/ml or 64/4 μg/L TZP, respectively, and incubated at 37°C for up to 5 days. TZP-resistant *Escherichia coli* strain 907355 was used as a positive control (Schechter et al., 2018). This method will not identify anaerobes or susceptible strains that were protected by other mechanisms when in the gut microbial community.

### Quantitative PCR

Quantitative PCR was carried out using SYBR Green reagents and protocols (Green and Sambrook, 2018). DNA was first diluted to between 0.001 and 1.0 ng/μl to optimize detection of differences in bacterial loads between samples. The master mix of SYBR Green reagents and primer was created by combining 12.5 μl of SYBR Green and 7.5 μl of primer for each reaction. A total of 20 μl of the master mix was added and 5 μl of the diluted DNA was added to each well of the reaction plate to make a total reaction volume of 25 μl. Primers used in these experiments are found in Table 1 and target the 16S rRNA gene. The Viia 7 Real-Time PCR machine (Life Technologies, Carlsbad, CA, United States) carried out the reaction with SYBR Green Fast reagents and comparative Ct analysis. Each qPCR was run with 40 cycles, with each cycle running at 95°C for 1 s and 45°C for 45 s.

**Table 1:** Quantitative PCR (qPCR) primers utilized in this study.

	Forward primer 5'–3'	Reverse primer 5'–3'	Citation
Universal	TCCTACGGGAGGCAGCAGT	GGACTACCAGGGTATCTAATCCTGTT	Nadkarni et al., 2002
Bacteroidetes	CRAACAGGATTAGATACCCT	GG TAAGG TTCCTCGCGTAT	De Gregoris et al., 2011
Firmicutes	TGAAACTYAAAGGAATTGACG	ACCATGCACCACCTG TC	De Gregoris et al., 2011
Gammaproteobacteria	TCGTCAGCTCGTGTYGTGA	CGTAAGG GCCATGATG	De Gregoris et al., 2011
OTU-1 Clostridiales	GGTTTGAAGGCATCTTCTTTCC	GTTAGCCGAGGCTTATTCCTAA	This Study

*Primer names, forward, and reverse sequences are shown for each primer set. Relevant publications are listed for all except the operational taxonomic unit (OTU)-1 Clostridiales primers, which were designed for this study*.

### Mass Spectrometry Sample Preparation

Pelleted mouse feces were shipped on ice to the mass spectrometry facility, where they were stored at −20°C. Samples were then lyophilized using a Labconco Rotovap, and dry feces were weighed using a Metler Toledo XPE206 analytical balance. Post-lyophilization, samples were returned to the freezer until extraction. Several extraction solvents were tested including 100% methanol (MeOH), 40% MeOH-40% ACN-20% distilled water, and 80% MeOH-20%ACN in 1.5 ml glass bead vials. Neat methanol was found to be the best, so the lyophilized mouse feces were homogenized using 100% MeOH. Homogenization was performed manually using a pestle and mortar procedure. Extraction volume was added at 50 mg/ml of feces/MeOH. After extraction, sample vials were centrifuged at 14,000 rpm for 5 min to settle particles. Aliquots of 100 μl supernatant were placed in a clean 1.5 ml vial. Samples were then rotovapped to dryness at 45°C for 60 min, then reconstituted in 100 μl of 0.1% formic acid water with 500 ng/ml reserpine for QTOF analysis (see below).

### Instrumentation

Samples were analyzed by a HPLC Quadrupole Time of Flight mass spectrometry (QTOF MS) applying Electrospray Ionization (ESI). Separation was performed by reverse-phase chromatography using an Agilent 1260 instrument (Santa Clara, CA, United States). MS analysis was carried out by a SCIEX QTOF 5600 mass spectrometer with a Sciex DuoSpray™ ion source (Concord, Ontario Canada). The auto-sampler was operated in partial loop injection mode with an injection volume of 10 μl. After injection, LC flow was bypassed for 3 min with a VICI bypass valve (Houston, TX, United States) before switching to the analytical column. For positive ion mode acquisition, analytes were eluted using a 2%–90% gradient organic solute in 17 min followed by a 5 min hold at 90%, then a 6 s return to 2% organic holding for 5 min to re-equilibrate the column. A flow rate of 0.2 ml/min was employed using 0.1% formic acid/water for the aqueous phase and 0.1% formic acid/ACN for the organic phase. For negative ion mode acquisition, analytes were eluted using a 2%–90% gradient organic in 9.5 min followed by a 150 s hold at 90%, then a 6 s return to 2% organic, held for 144 s to re-equilibrate the column. A flow rate of 0.5 ml/min was achieved using 5.4 mM ammonium acetate-water for the aqueous phase and ACN for the organic phase.

Mass spectra and tandem mass spectra were recorded in both positive- and negative-ion modes with the “high-sensitivity” mode selected. A TOF scan from 75 to 1,000 Daltons (Da) was performed involving four time-bins and all four time-to-digital converter (TDC) channels selected. Information dependent acquisition (IDA) was set to trigger MS/MS product ion scans when the signal exceeded 100 counts/s (CPS), whereas dynamic accumulation was on and a maximum of six dependent candidate ions were scanned per cycle.

### HPLC QTOF MS Run Sequence

A typical run sequence began with two double blank injections to condition the system, followed by six consecutive injections of 500 ng/ml reserpine to define system suitability. Sequence order of sample analysis was randomized using Microsoft Excel. Instrument calibration was performed every 10 samples (5 h) using the Sciex Calibration Delivery System with Sciex APCI positive calibration solution part #4460131 for positive mode acquisition and Sciex APCI negative calibration solution part #4460134 for negative mode acquisition.

### Full-Length 16S Sequences

Fecal samples were selected from mouse 6, Day 9 from TZP1, and mice 9 (immune-competent) and 19 (neutropenic), Day 18 from TZP2 in which the Clostridiales OTU (OTU-1 and -3 in TZP1 and TZP2, respectively) was present at >95% relative abundance. DNA was extracted from stool using a Qiagen extraction kit (QIAGEN, Hilden, Germany), and 1 μg DNA was used as template to prepare SMRTbell libraries. DNA was sheared using Covaris g-tubes at 4,000 rpm for 3–4 min and cleaned using 0.75X AmPure XP beads to size select the desired range of fragments and remove all DNA fragments <200 bp. PacBio sequencing libraries were prepared using the SMRTbell template prep kit 1.0 (Pacific Biosciences, Menlo Park, CA, United States) according to the manufacturer’s protocol. Quality assessment of libraries was performed using the Qubit Fluorometer (Thermofisher) and the TapeStation (Agilent, Santa Clara, CA, United States). Loading protocols for each sample were made using PacBio SMRTLink v 6.0. Libraries were annealed to Conditioned Sequencing Primer v3.0 then bound to Sequel Polymerase v3.0 in accordance with SMRTLink directions. Libraries then underwent a Qubit, AMPure PB cleanup and a second Qubit run. Based on the final Qubit analysis, libraries were diluted and Internal Control v3.0 was added to each sample. Final libraries were loaded onto the Sequel according to SMRTLink directions. Libraries were run at 6 pm with Sequencing Kit 3.0 V2 and SMRTcells v3.0. Immobilization time was set at 120 min, Pre-Extension was set at 120 min and Movie Time was set at 600 min for each SMRTcell. Output files were processed and assembled into circular consensus sequencing (CCS) reads using PacBio Sequel SMRTLink-Release_7.0.0.63985 software default settings with minimum passes to 3 and minimum predicted accuracy to 0.9. Sequence orientation was assessed and, if necessary, corrected using the orient command in USEARCH v10.0.240 with the GreenGenes database v13_5 as a reference. Sequences between 900 and 1,800 bp were kept for further analysis.

### Sequence Cleaning and Annotation

Initial examination of sequence data indicated very high sequence diversity (even in samples that were assumed to contain very few taxa). Therefore, to account for the possibility that such diversity was driven by sequencing error, additional filtering steps were applied as described hereafter. Sequences were initially processed separately for data from mouse 6, but combined for data generated from mice 9 and 19 as their 16S rRNA gene V1–V3 region sequences clustered into the same 97% OTU. Sequences from each group were de-replicated and clustered into OTUs with 97% similarity using USEARCH fastx_uniques and cluster_otus commands with default settings.

Reads from each group (mouse 6, mice 9/19) were then aligned to the representative sequences for their respective OTUs using cross_match v1.090518 and rates of substitution, deletion, and insertion errors calculated. Total error was calculated by the substitution, deletion, and insertion errors. Using an exponential decay function, CCS pass numbers were modeled against the total error rate and a CCS pass cutoff that gave less than a 2.3% error rate for the 95th percentile of the data was selected. Sequence reads below this CCS pass threshold were subsequently removed from each group. Data from mouse 6 required nine CCS passes to get 95% of the data to below 2.3% total error while mice 9 and 19 required seven CCS passes.

### Phylogenetic Tree Placement

In order to identify the likely taxonomy of the V1–V3 Clostridiales OTUs present after antibiotic treatment, each OTU was first matched to full-length 16S rRNA gene sequences generated as described above. Subsequently, a phylogenetic tree was built using matched PacBio full-length 16S rRNA gene sequences and their closest full-length 16S homologues present in the NCBI 16S Refseq database. Full-length PacBio 16S rRNA gene sequences remaining after the CCS pass filtering described above were pooled to combine the sequences generated from mouse 6 with those generated from mice 9 and 19. Pooled sequences were then de-replicated and filtered using the USEARCH “derep_fulllength” command to retain only unique sequences represented five or more times. Remaining sequences were indexed using the “makeblastdb” command in BLAST+ v2.6.0. Representative V1–V3 OTU Clostridiales sequences were searched against the resulting databases to identify all occasions where a V1–V3 sequence was an exact match to one or more full-length 16S sequences. In total, 10 full-length 16S gene sequences matched the V1–V3 OTUs with 100% identity across 100% of the V1–V3 sequence.

### Identification of the 16S rRNA Gene OTU

In order to identify the likely taxonomy of the 10 candidate full-length 16S rRNA gene sequences, each sequence was aligned to the NCBI 16S RefSeq database (download 27 June 2019) using the “blastn” (BLAST+ v2.6.0) command with the option “max_target_seqs” set to 500 (Madden et al., 2019). Matching target sequences were then sorted by *e*-value, followed by bitscore, and the top 100 best matches for each query sequence were combined. After removing duplicate matches, this resulted in 182 unique NCBI 16S RefSeq sequences with homology to one or more of our 10 query sequences. The 182 RefSeq 16S rRNA gene sequences and 10 query sequences were pooled and aligned against the GreenGenes reference database using PyNAST v0.1 (Caporaso et al., 2010). PyNAST was run using the QIIME (v1.9.0) script “align_seqs.py” with a minimum of 75% identity. During PyNAST alignment of the RefSeq 16S sequences, one sequence had only ~83% mean identity to the reference alignment and was removed from further analysis. The resulting alignment was visually assessed on SeaView v4.7 (Gouy et al., 2010; Carretero-Paulet and Albert, 2016) during which nine additional RefSeq 16S rRNA gene sequences were removed due to disproportionately short length or poor alignment. The remaining 182 sequences (including the 10 sample sequences) were trimmed at positions 68–1,907 and empty columns were removed by Splicer v3 (Srivastava et al., 2017). Aligned sequences were subsequently processed using the QIIME script “filter_alignment.py,” using a gap filter threshold of 0.95. Finally, a Neighbor-Joining tree was constructed using FastTree v2.1.3 (Price et al., 2009) *via* the QIIME script “make_phylogeny.py.” The tree was visualized using the ggtree package v1.14.6 in R v3.5.1 (R Core Team, 2013; Yu et al., 2018).

## Results

To understand the effects of antibiotic treatment on the gut microbiome, two experiments were conducted in which TZP was administered to mice in a dosing regimen designed to mimic the human clinical dosing levels (Bulik et al., 2012). TZP1 consisted of 6 days pre-TZP monitoring (including 48 h of acclimation after arrival to the vivarium from the vendor), 7 days of TZP treatment, and 7 days post-TZP treatment follow-up. After conducting preliminary analyses on the fecal pellets from this experiment, we designed a second experiment, TZP2, in which two sets of 10 mice, immunocompetent as well as neutropenic, were allowed a 14-day pre-TZP time period, to observe bacterial community changes during the acclimation period, followed by 4 days of TZP exposure using the humanized dosing regimen, and 21 days of post-antibiotic recovery period. The neutropenic model is important since TZP is often used a therapeutic agent in neutropenic human patients.

### Assessment of Gastro-Intestinal Bacterial Loads Before, During, and After TZP Treatment

**Figure 1:**
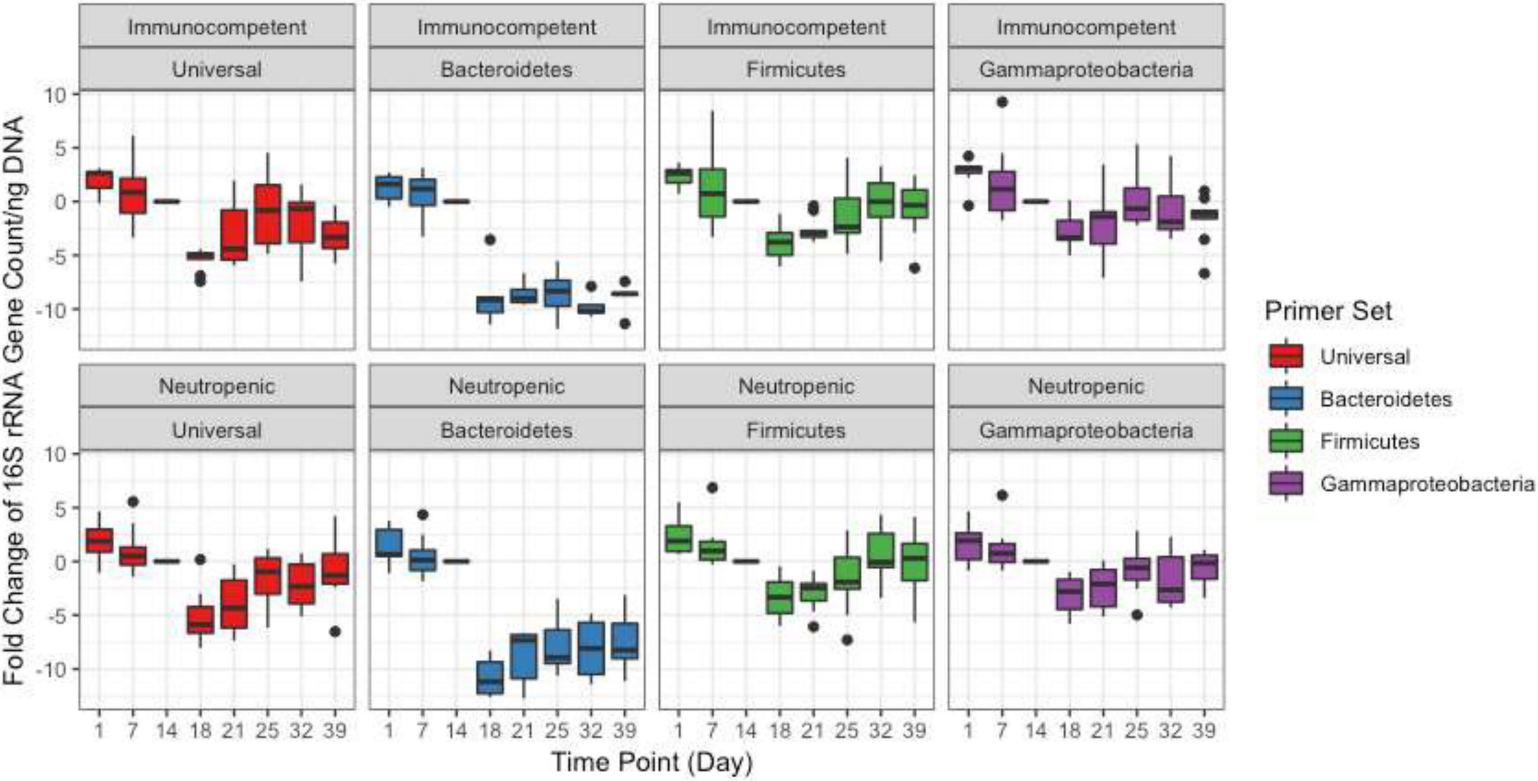
Bacterial quantification of the TZP2 experiment. Box and whiskers plots show the fold change of the 16S rRNA gene count as generated by quantitative PCR (qPCR) from DNA extracted from stool for all mice (immunocompetent and neutropenic shown separately) at each time point as compared to Day 14, the time-point immediately prior to antibiotic treatment.

Quantitative PCR was performed to quantify the bacterial load using universal 16S rRNA primers as well as phylum-specific primer sets (Table 1; Figure 1; Supplementary Figure 1; Nadkarni et al., 2002; De Gregoris et al., 2011). Bacterial loads for each mouse at each time point were compared by calculating the fold change in 16S rRNA amplicons with respect to the last pre-antibiotic time point (day 6 and 14 in TZP1 and TZP2, respectively). The overall bacterial loads in both experiments decreased upon antibiotic exposure, up to 5–10-fold, and began to increase again upon termination of the antibiotic administration, though the bacterial loads in both experiments did not return to their respective pre-antibiotic levels. The Bacteroidetes-specific primers showed that Bacteroidetes decreased up to 10–15-fold upon exposure to TZP and remained low even 2 weeks after termination of TZP, possibly indicating more permanent changes. Firmicutes and Gammaproteobacteria were the first to recover in terms of bacterial load. The dynamics observed here suggest different antibiotic-induced scenarios for different phyla of bacteria. Of note, there do not seem to be major differences at this level between immune-competent and neutropenic mice.

### Changes in Bacterial Community Composition

**Figure 2:**
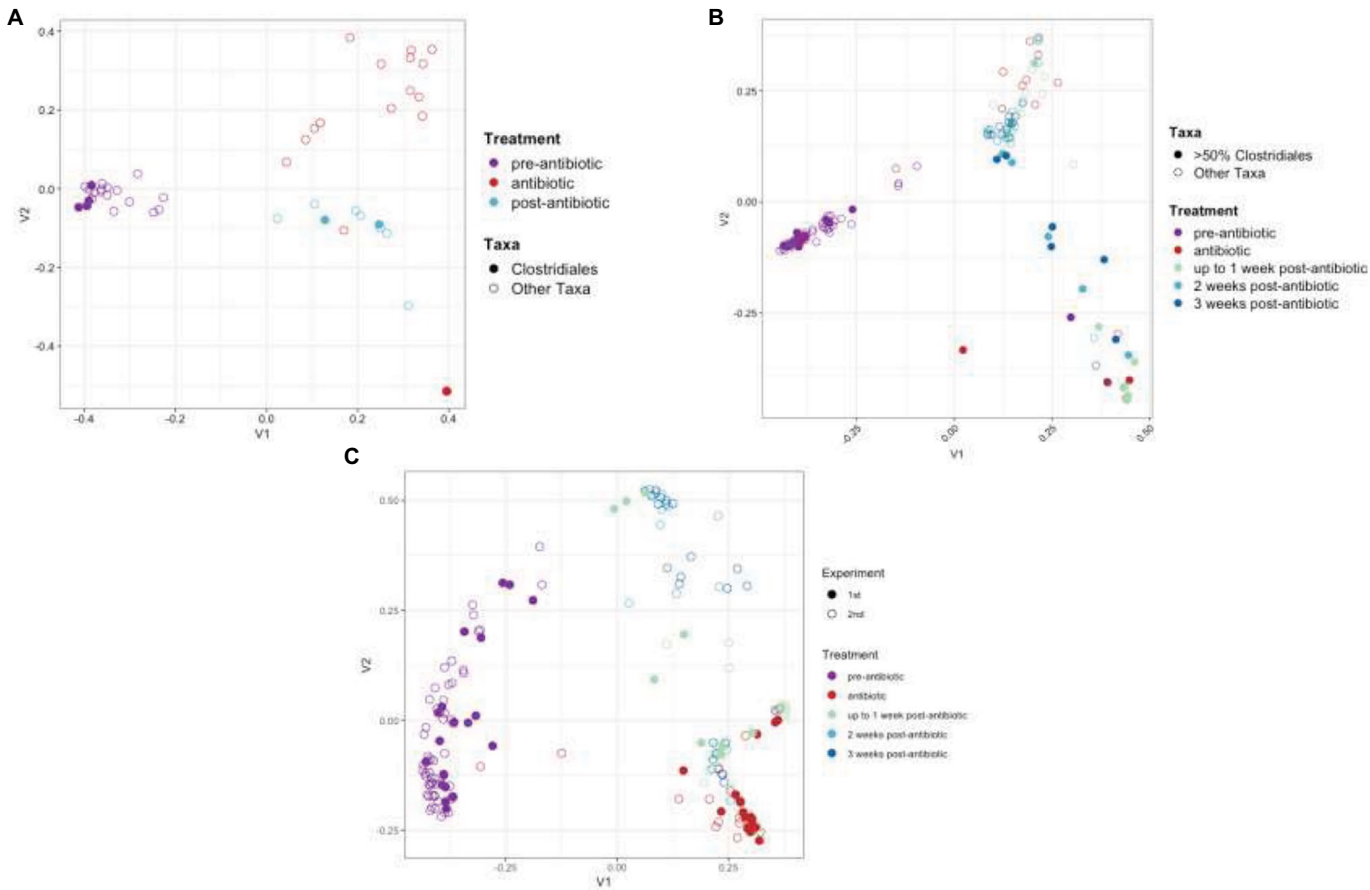
Principal coordinate analysis (PCoA) of taxonomic profiles. PCoA analysis based on the Bray-Curtis distance matrix of OTUs from TZP1 **(A)** and TZP2 **(B)** experiments. Each circle represents a single mouse at a single time point. Closed circles represent mice that had >50% Clostridiales (OTU-1 in **A**, OTU-3 in **B**) at any time point and open circles represent all other mice. **(C)** PCoA analysis on Bray-Curtis distance matrix based on combined genera profiles from both experiments. Note that the number of bullets per panel may vary as a consequence of the number of mice per group and the number of samples taken and analyzed in full per individual mouse.

The bacterial communities were monitored by targeted sequencing of the V1–V3 variable regions of the 16S rRNA genes. Key time points were chosen before, during, and after TZP administration. Sequence reads were clustered into OTUs based on at least 97% sequence identity. The relative abundance of these OTUs formed a taxonomic profile for each time point for each mouse. A principal coordinate analysis (PCoA) based on the Bray-Curtis distance matrix shows the similarity of the bacterial communities in each mouse for each time period, and how they shifted in response to the antibiotics and during the recovery period (Figures 2A,B). The pre-TZP time points are tightly clustered together, indicating that all mice had very similar microbiomes prior to antibiotic exposure. Upon antibiotic exposure, the taxonomic profiles split into two clusters. The cluster at the bottom right of the graph consists of mice that had 50%–100% relative abundance of a single OTU that was classified in the order Clostridiales, but did not match sequences in databases at lower phylogenetic levels. Mice that fit this profile are indicated by a closed circle. All other mice (<50% Clostridiales) are indicated with open circles, and the taxonomic profiles for the antibiotic time point (red) for those mice cluster in the upper right portion of the graphs. As the mice entered the recovery period, the Clostridiales-dominant and other mice clusters began to shift closer together again, indicating a more similar profile.

As the OTU clustering was performed separately for each experiment, a joint analysis of the two experiments was performed at the genus level, using the taxonomic classifications assigned to each OTU using RDP classifier. Figure 2C shows a joint PCoA plot of both TZP1 and TZP2 based on the Bray-Curtis distance matrix of their respective genera profiles. For each pre-, during, and post-TZP period, the graph shows that the three treatments clustered separately. However, the closed (TZP1) and open (TZP2) circles are intermingled, showing that the taxonomic profiles and their antibiotic-induced shifts are reproducible.

**Figure 3:**
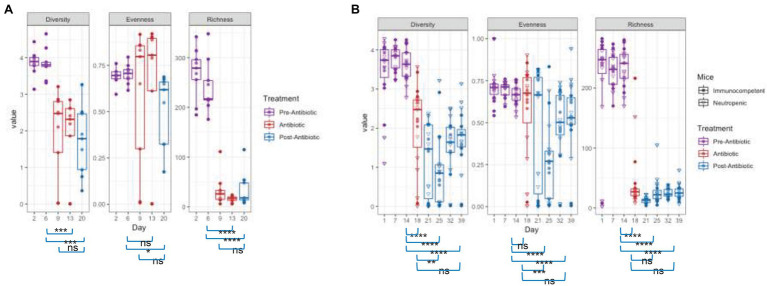
Effect of piperacillin-tazobactam (TZP) on the Alpha Diversity. The alpha diversity of each mouse in each time point was measured using Pielou’s evenness, absolute number of OTUs (richness), and Shannon diversity index (diversity). Significant differences between two time points are indicated by asterisks where *p* > 0.05 = ns, *p* < 0.05 = *, *p* < 0.01 = **, *p* < 0.001 = ***, and *p* < 0.0001 = ****. Panel **(A)** shows results obtained during the TZP1 study, whereas panel **(B)** displays the results obtained during the TZP2 study.

The changes in community structure of the combined data sets were measured for each time point, assessing richness (total taxa), evenness (uniformity of abundances of taxa), and using the Shannon diversity index, which considers both evenness and richness (Figure 3). Upon antibiotic exposure, the diversity dropped significantly (*p* = 0.0012 between days 6 and 13 for TZP1 and *p* = 2.83 × 10^−6^ between days 14 and 18 in TZP2, using Fisher’s T-Test). The diversity continued to decrease within 1 week after TZP treatment (*p* = 0.0002 between days 14 and 20 in TZP1 and *p* = 7.22 × 10^−10^ between days 19 and 25 in TZP2), and began to recover 2 weeks post-TZP treatment in TZP2, though still significantly different from the pre-TZP time point (*p* = 6.77 × 10^−8^). Diversity recovery was not observed in TZP1 as it was terminated 1 week post-TZP. The evenness of the taxa prior to TZP treatment was tightly associated, with all mice having similar evenness. During and post-TZP treatment, the evenness was more widespread across the different mice, with some having less evenness than others. Still, a significant decrease in the mean evenness was observed, as expected if dominant organisms emerged, with the lowest mean occurring at 1 week post-treatment (*p* = 0.0232 and 7.22 × 10^−10^ between days 15 and 20, and 19 and 25, in TZP1 and TZP2, respectively). Richness significantly decreased upon antibiotic treatment, consistent with the decrease in bacterial load, and remained significantly lower than the pre-antibiotic state up through the end of TZP2, 3 weeks post-antibiotic (*p* = 3.80 × 10^−15^ between days 19 and 39). As noted above, there do not seem to be significant differences between immune-competent and neutropenic mice.

### Changes in Overall Community Structure

**Figure 4:**
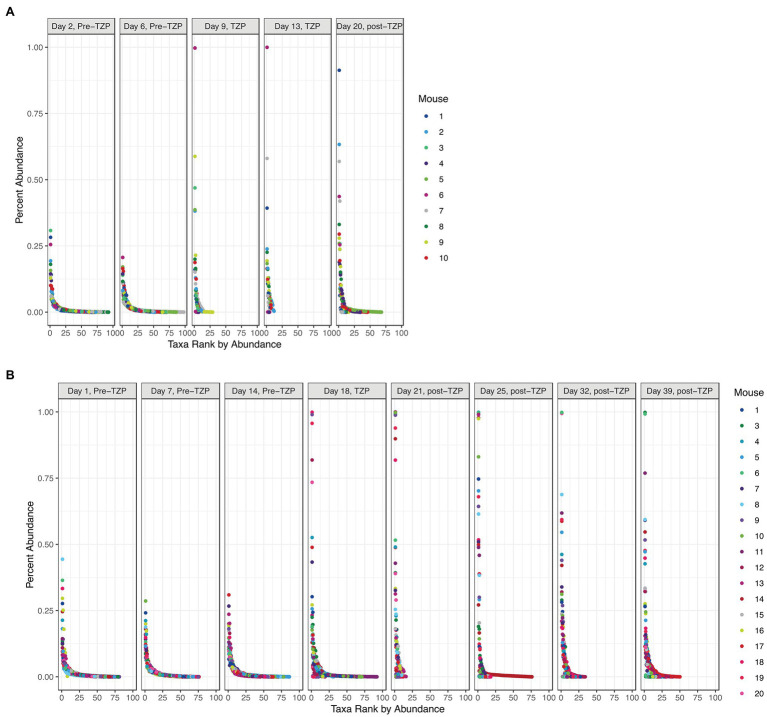
Bacterial community structure. The OTUs for all mice in each experiment (TZP1 in **A**, TZP2 in **B**) are plotted by ranked abundance for each time point.

In addition to significant changes in alpha diversity and taxonomic profiles, we also observed changes to the overall community structure. Plotting the OTUs by their ranked abundance, the pre-TZP time points exhibited a community structure with all taxa at less than 0.5% abundance and over 75 taxa detectable for ranking (Figure 4). Upon TZP treatment, the community shifted dramatically with some taxa present at 0.5%–1% abundance and less than 30 ranked taxa, similar to the observations for OTU richness. In the immediate post-TZP time points, some mice experienced shifts in their microbiota with more ranked taxa present, although this trend appeared in few mice and was unstable in the later time points during recovery. These results may indicate a fluctuating community structure where a dominating organism may change over time, as reported in environmental samples (Ofiteru et al., 2010).

### Species Composition

**Figure 5:**
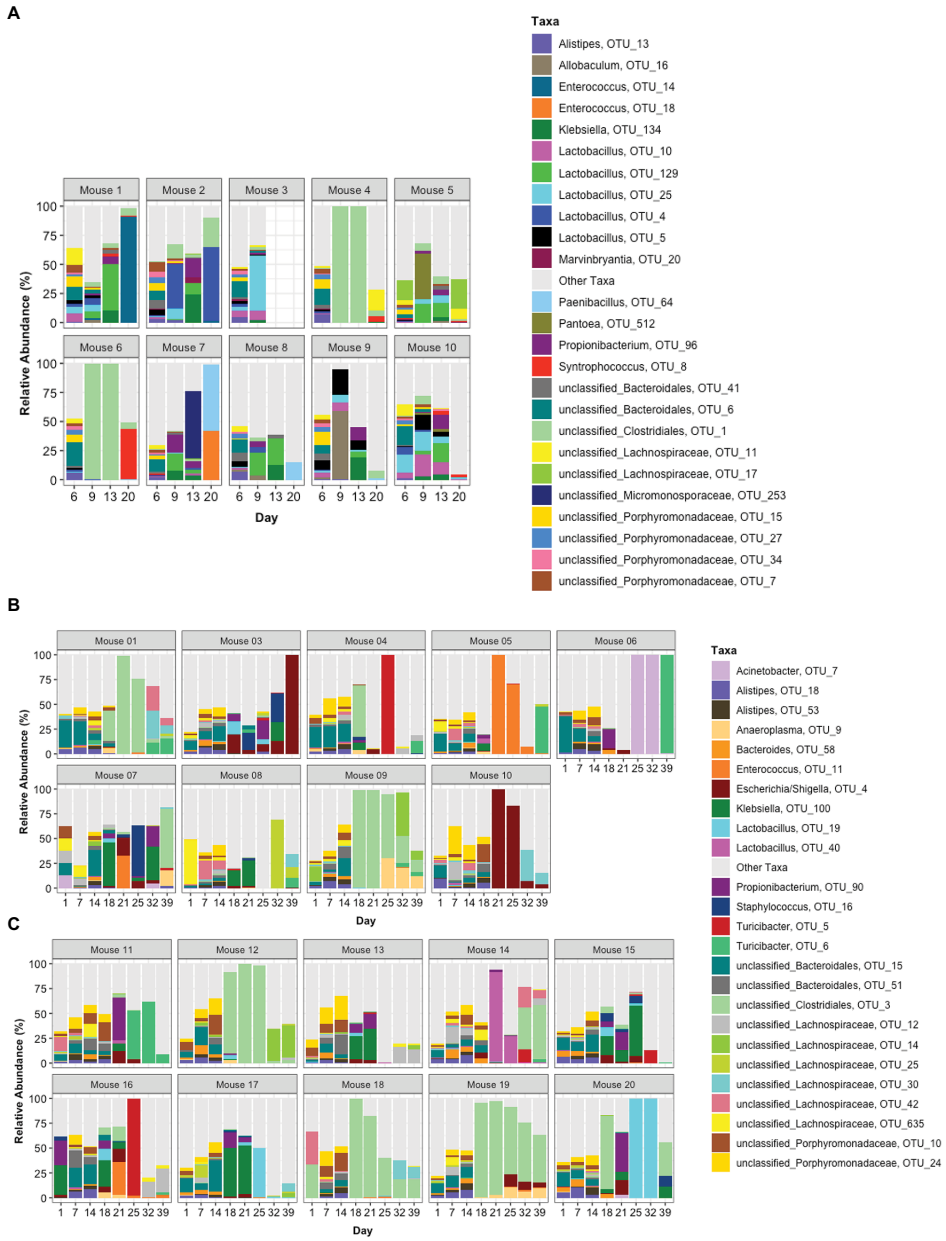
Taxonomic profiles. The relative abundance of taxa is represented by stacked bars for each mouse and each day. The top 25 OTUs are colored individually and all other OTUs are assigned to the “Other Taxa” group (light gray). **(A)** Portrays TZP1 in which TZP was administered days 7–13, **(B,C)** portray immunocompetent mice (mice 1–10) and neutropenic mice (mice 11–20), respectively, from TZP2 in which TZP was administered on days 15–18.

The top 25 taxa are plotted in Figure 5A to show the relative abundance of each taxon at each time point for each mouse. Taxa composition in the pre-TZP time points was more diverse than during and immediately after the TZP treatment. A single OTU was predominant in some mice during and post-TZP treatment. The microbiota in several mice were dominated by unclassified Clostridiales (OTUs 1 and 3 in Figures 5A–C, respectively; for similar data obtained for TZP1 see Supplementary Figures 3A–C, 4).

**Figure 6:**
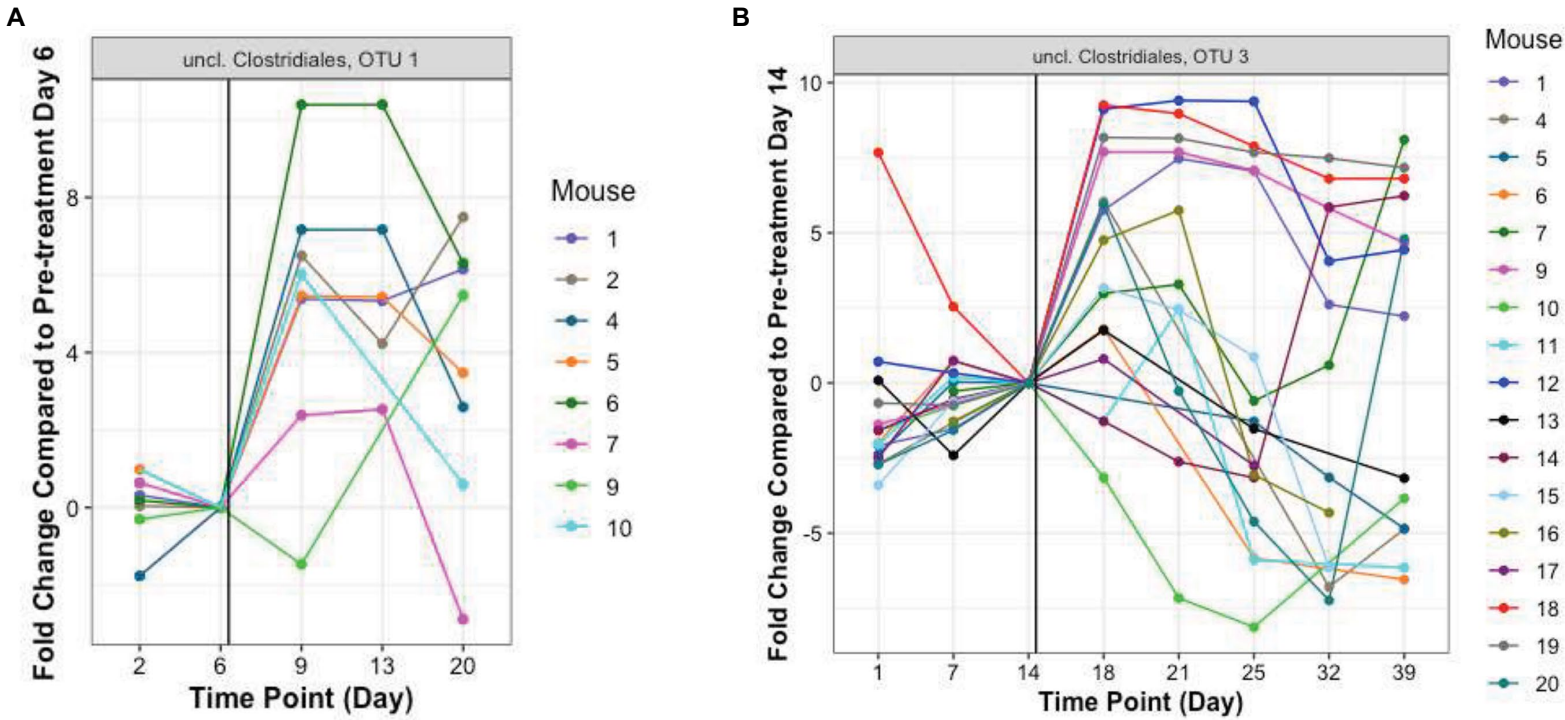
Resistant Clostridiales OTUs. The fold change of relative abundance of Clostridiales OTUs 1 and 3, (**A,B**, TZP1 and TZP2, respectively) compared to the last pre-antibiotic time point (Days 6 and 14, respectively) are plotted for each day. The vertical line indicates the approximate first TZP exposure. Mice 1–10 were immunocompetent while mice 11–20 were neutropenic.

**Figure 7:**
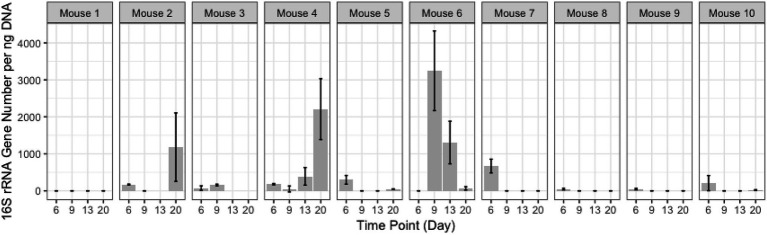
OTU-1 Abundance. Absolute abundance of OTU-1 in TZP1 estimated by targeted qPCR.

In addition to these proportionately dominant taxa, we studied if any minor organisms showed enrichment after antibiotic treatment. To determine if the antibiotic treatment selected for or promoted the growth of certain organisms, we calculated, for each OTU, the relative abundance during the antibiotic treatment and immediately after treatment, compared to the last pre-TZP time point. There were 15 OTUs that increased in one or two mice (Supplementary Figures 5A,B); OTUs 1 and 3 increased in many more mice (Figures 5B, 6). Since these data only show the relative abundance and do not indicate whether the bacteria were in fact increasing in absolute numbers during these time points, qPCR was performed on samples from TZP1 with custom primers specific to the OTU-1 V1–V3 region (Figure 7). The bacterial load of this OTU increased in several mice, including mouse 6, one of the two mice that had ~100% relative abundance of this OTU during days 9 and 13. However, the absolute abundance of this organism was in general low, and difficult to accurately quantify. We were not able to grow these bacteria when samples from TZP1 were cultured on TZP chromID plates, most likely since Clostridiales are anaerobic and non-capable of growing in the presence of excessive oxygen. Subsequent efforts to recover these bacteria from frozen stool pellets were also unsuccessful.

### Full-Length 16S rRNA Sequencing for Predominant Species

**Figure 8:**
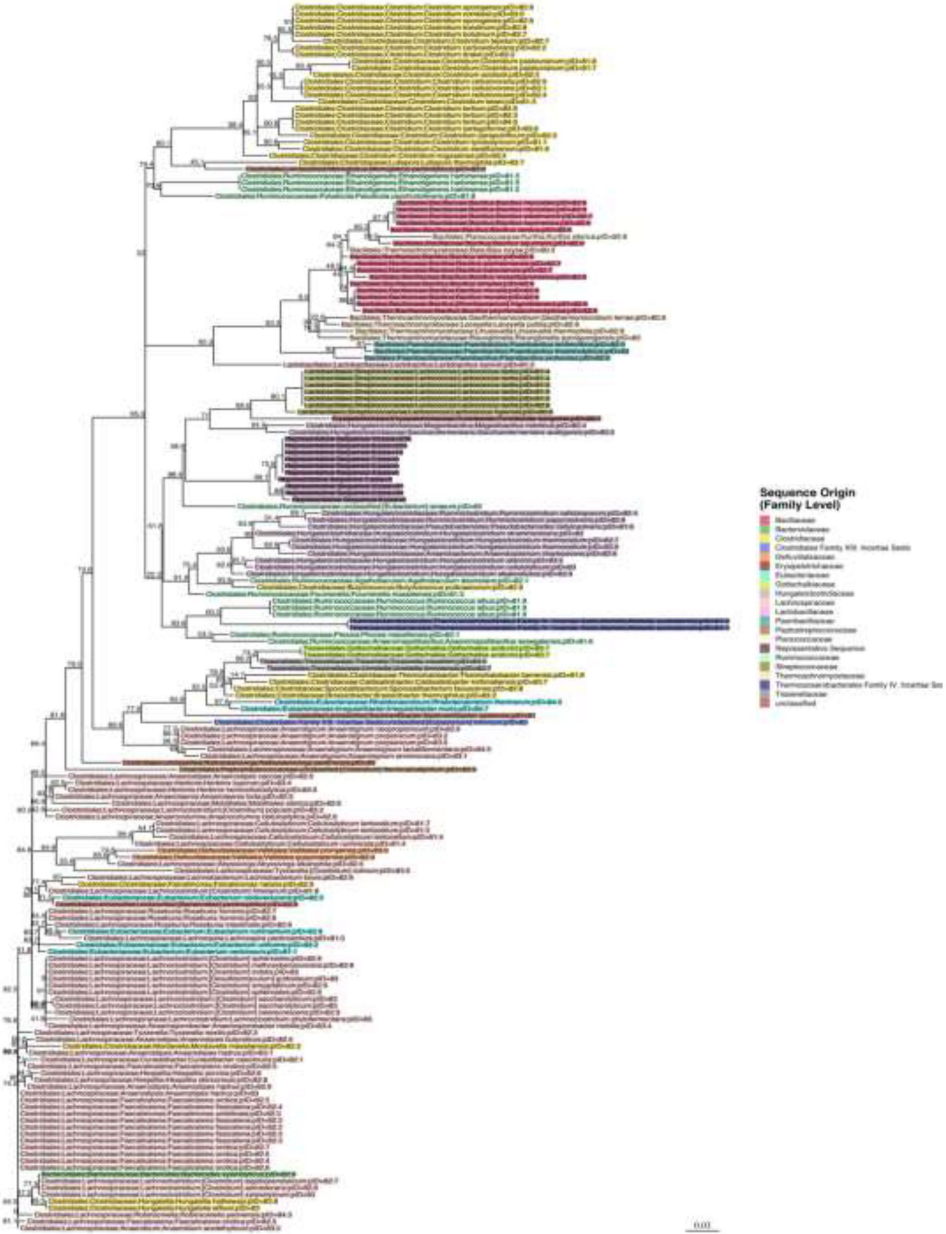
Phylogenetic tree for the newly discovered dominating species in some of the mice post antibiotic treatment.

Representative sequences from the top 10 OTUs from three mice per sequence motif were all highly similar. Bootstrap values are shown next to each node in the phylogenetic tree that was built around these 16S sequences with each label colored by family (Figure 8). The representative sequences of the top 10 full-length 16S rRNA gene OTUs cluster together in the middle of the tree (labeled as representative sequence 1–10). Surrounding reference sequences were the best BLAST hits from GenBank’s RefSeq collection and the labels include the order, family, genus, and species. It appears that the organism belongs to the Clostridiales order, in the Hungateiclostridiaceae family (Zhang et al., 2018). This family is newly proposed for the clade of Clostridium 16S III taxa that were previously in the Ruminococcaceae family but are phylogenetically distinct. Given its branch length and low percent identity to other taxa, we believe this is a new genus and species that has not yet been described. Its closest matches are *Eubacterium siraeum* (branch below) and *Mageeibacillus indolicus* and *Saccharofermentans acetigenes* (branch above). *Eubacterium siraeum* has brackets around the genus name because NCBI noted that the genus is still awaiting reassignment. It is thought that this taxon belongs to the Hungateiclostridiaceae family, mostly isolated from the human GI tract. The *M. indolicus* and *S. acetigenes* were both recently discovered spore-formers from the vaginal microbiome (Austin et al., 2015).

### Immune-Competent vs. Neutropenic Mice

The possibility that the microbiota shift in response to the antibiotic might differ in immunocompetent and neutropenic mice was studied. A PCoA plot based on the Bray-Curtis distance matrix of the OTU profiles for each mouse in TZP2 was generated with the closed circles representing immunocompetent mice and open circles representing neutropenic mice (Supplementary Figure 2). At each of the pre-, during, and post-TZP time points the closed and open circles are intermingled, showing that the cyclophosphamide-affected immune status of the mice does not appear to have a major effect on the microbiota changes. This leads us to conclude that the immediate, short-term effect of cyclophosphamide on the gastro-intestinal flora of mice is apparently negligible. Longer term effects have not yet been assessed here but obviously warrant additional studies.

### Piperacillin Levels in the Fecal Samples

The level of piperacillin in the feces was determined by HPLC of samples from each mouse on days 13 and 20 for the first experiment, and days 15, 18, 19, and 20 for the second experiment (Supplementary Figures 6, 7). Experiments were performed to confirm the concentration as well as track the levels of the piperacillin post-treatment. Between 2 and 7 days after discontinuation of treatment, the piperacillin concentration generally decreased by a factor of 1,000 on average.

### Metabolomic Analyses

**Figure 9:**
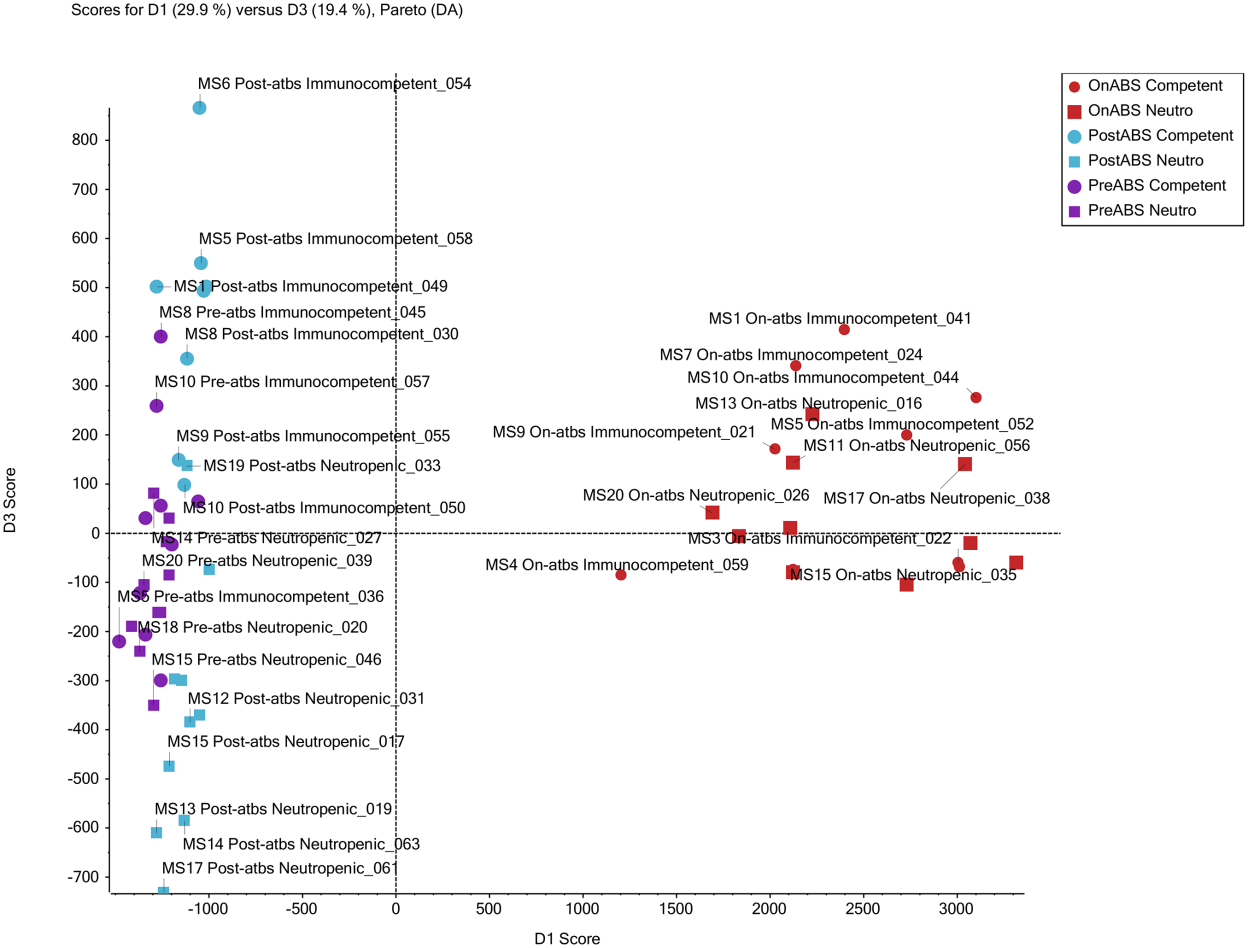
Metabolomic profiling. Principal component analysis of non-targeted TripleTOF mass spectrometer (MS) data of mouse feces successfully grouped treatment from non-treatment mice. The scores plot for PC1 and PC3 of MS features in positive ion mode shows good separation of the antibiotic vs. non-antibiotic time points. Furthermore, clusters of post-antibiotic immunocompetent and post-antibiotic neutropenic features also show good separation from each other with immunocompetent clustering above and neutropenic below the *x*-axis.

Supervised PCoA score plots without weighting and Pareto scaling were performed on the metabolomics data. Score plots for PC1 and PC2 of the overall QTOF MS features, found in positive ion mode, show good separation of on-TZP vs. off-TZP animals (red symbols in Figure 9). The on-TZP samples clustered together for both immune-competent and neutropenic mice, again showing the lack of significant differences between the groups as was also demonstrated at the bacterial taxon levels. The pre-TZP and post-TZP samples were grouped in the left quadrant of the plot with post-TZP components in the upper left and the pre-TZP in the quadrant below separating the two treatment groups. The pre-TZP and post-TZP components also correlate well on a linear basis. Score plots of PC1 and PC3, defined in negative ion mode show similar patterns but with more specific clustering of both treatment groups. Spectral features that cluster due to TZP exposure were captured while comparing the pre-TZP group with the other sample groups. Score plotting of PC3 and PC4 quantifies the variance between immune-competent and neutropenic mice, which again is non-significant. In conclusion, metabolomic analyses were performed on the fecal pellets from the second experiment mostly to look for changes in metabolites. Metabolomic profiling of each mouse showed that the metabolomes of mice pre-TZP were similar and the metabolomes during TZP exposure were also similar (Figure 9). The post-TZP metabolomes of mice were separated based upon whether the mouse was immune-competent or neutropenic, something the bacterial taxonomy tests could not. Overall, there is good congruency between taxonomic and metabolomic analyses. Currently, it is unclear which fraction of the metabolomic markers is of microbial vs. human origin. Also, we have not been able to detect and quantify short chain fatty acids (SCFA) and bile acids, molecules known to be of eminent biological relevance in microbiome modelling. Further studies into the relevance of these two categories of molecules on the dynamics of our model are still required.

## Discussion

Further data assembly and interpretation is needed to identify the precise bacterial species and associated metabolomic features, which play major roles in the antibiotic response in gut microbiome. Many medical treatments have a major effect on the composition of the human microbiome (e.g., Minalyan et al., 2017 or Shukla et al., 2019). This mostly involves antibiotics but other commonly used drugs may produce distinct effects as well (Maier et al., 2018). Whether such disturbances are short-term and whether microbiomes return to their original state after antibiotic treatment are currently ill-defined (Dethlefsen and Relman, 2011; Nobel et al., 2015). In rat and mouse models, however, it has been demonstrated that changes in the microbiome depend on the type of antibiotic (Tulstrup et al., 2015) and the microbiome composition may pre-dispose toward susceptibility to certain types of controlled infection (Theriot et al., 2014; Koo et al., 2019). Volunteer studies in humans showed that, in part, depending on the initial status of GI flora diversity, common species may disappear upon antibiotic treatment and not recover (Dethlefsen et al., 2008; Raymond et al., 2016a,b; Palleja et al., 2018). TZP treatment in humans undergoing hematopoietic stem cell transplantation caused severe declines in cell counts for gastro-intestinal obligate anaerobes (Morjaria et al., 2019). Although these studies concluded that bacterial diversity was usually restored relatively quickly after treatment, there is continuing lack of clarity as to the short-term effects of enrichment of resistant organisms in the GI tract and the long term effects of the remaining flora changes (Roodgar et al., 2021). Jakobsson et al. (2010), for instance, were able to show that anti-*Helicobacter pylori* treatment may have consequences on the flora of the throat and GI that may last for years and lead to enrichment of resistance genes. This finding reveals a serious side effect of antibiotic usage, as resistance genes can cross species barriers while residing in the GI flora (Shoemaker et al., 2001). In specific patient cohorts, personalized prediction of the effect of antibiotic treatment will require combined interpretation of microbiome, resistome, and even mobilome data (Willmann et al., 2019). We here show reproducible restoration effects in individual mice after TZP treatment on the composition and bacterial density of the gastro-intestinal flora in a standardized mice model of antibiotic treatment. Still, we do observe heterogeneity in the flora restoration patterns between different mouse individuals, although less than among humans. Hence, we feel that the model proposed here is going to be helpful in defining early detrimental effects of antibiotic treatment and their later effect on restoration of flora diversity. Different antibiotics can be used in combination with different mouse genotypes whereas also external factors including diet can be modulated contemporaneously.

### The TZP Model

We here define a non-infectious mouse model potentially suited for extrapolation regarding the use of antibiotics in humans since we use a humanized dosing schedule. Still, TZP is associated with a complicated variety of selective features. It selects for beta-lactamase hyper-producers and may help enrich multiple beta-lactamase types. The simple switch to alternative antibiotic classes will not easily result in reversion of resistance (Lee et al., 2013). Also, novel resistance mechanisms toward TZP are still being discovered (Schechter et al., 2018). Fortunately, we did not find obvious changes in phenotypic TZP resistance levels during our relatively short term experiments. Studies with other antibiotics and with longer treatment duration should shed more light on the emergence of resistance. Also, attempts to cultivate new species using strictly anaerobic culture methods and media were unsuccessful.

The gastro-intestinal bacterial load dropped significantly upon antibiotic exposure, exhibiting a slight recovery post-antibiotic treatment. We observed that the flora changes during and after TZP treatment can be reproduced between experiments. This suggests that the model is robust, although we have been using limited numbers of mice thus far. In addition, TZP treatment pushes the flora into two dynamic directions, clearly visible in our diversity plots and partial ribosomal sequencing data. Two distinct taxonomic profiles were observed during and after antibiotic exposure, where mice had either predominantly Firmicutes or a more diverse mix of taxa in their feces. One of the profiles mentioned above mainly displays the dominant expansion of a single bacterial species, which is likely a new one that should be integrated into the Mouse Intestinal Bacterial Collection (Lagkouvardos et al., 2016). In several mice, this single species dominated the post-antibiotic flora. It would be interesting to study whether the different profiles lead to different health consequences post-treatment and whether or not restoration of the flora is equally feasible after either of the two format changes. All findings were reproducible between experiments.

### Metabolomic Variation Upon Antibiotic Treatment

The variation and dynamics of bacterial cell counts and taxa mentioned above can be noted at the level of the metabolome as well, showing an intricate association between the complexities of the microbiomes and the metabolomes (Nobel et al., 2015; Yachida et al., 2019). For that reason, coupled investigation on the diversity and dynamics of both entities are mandatory. Initial studies in a mouse model for *Clostridioides difficile* infection (CDI) already showed a tight correlation between changes in the gastro-intestinal flora and the presence and concentration of bile metabolites and low molecular weight carbon sources (Theriot et al., 2014). In humans, metabolomics allowed for the distinction of fecal metabolomes for individuals with CDI, patients with non-*C. difficile* diarrhea and those with mere *C. difficile* colonization (Robinson et al., 2019). Here we confirm a close correlation between the two also in our standardized mouse model. Further interpretation is needed to identify the precise MS-defined metabolome features and determine which specific molecules play a role in gut microbiome health and recovery after antibiotic treatment. Inclusion of the dietary composition has been shown to be important as well (Cabral et al., 2019) and our mouse model would allow detailed studies on the impact of changes in an otherwise homogeneous diet provided. Quantitative analysis can then be performed on the identified small molecules of interest to access potential biomarkers. Identification of the molecules, whether host-or microbiome-derived, may result in approaches that might help restore the GI flora in its initial format. It has already been demonstrated that the effects of antibiotic treatment in humans at the level of the metabolome may be more rapidly detectable than effects at the level of the microbiota (Vrbanac et al., 2020). This may have a significant impact on the development of microbial diagnostics reflecting changes in resident microbial flora in a range of eukaryotic hosts.

## Conclusion

We present a mouse model of humanized TZP treatment allowing for longitudinal screening of (changes in) GI microbiome diversity. We observe two major taxonomic profiles over time, one that is composed of more diverse flora and another one where a new bacterial species dominates the flora. The presence of this species results in a less complete restoration of the flora after TZP treatment. The bacterial variation over time is not significantly different between immune-competent and neutropenic mice, although metabolomic data differentiate between the two. The model is ready for further research into development of resistance and the analysis of long-term effects of the various microbiome evolutions on overall health of the model animals. Hopefully, data obtained in this standardized way can lead to extrapolation toward the human ecosystem and stimulate innovative translational research.

## Data Availability Statement

The datasets presented in this study can be found in online repositories. The names of the repository/repositories and accession number(s) can be found in the article/**Supplementary Material**.

## Ethics Statement

All murine experiments were executed in concordance with the National Research Council of the National Academy of Sciences standards. Study protocol was approved by the Institutional Animal Care and Use Committee of Hartford Hospital (Assurance #A3185-01).

## Author Contributions

All authors listed have made a substantial, direct, and intellectual contribution to the work, and approved it for publication.

## Conflict of Interest

AB, DB, WD, and LS are or were employees of bioMérieux SA (La Balme Les Grottes, France), a company developing, marketing, and selling tests in the infectious disease domain. The company had no influence on the design and execution of the clinical study neither did the company influence the choice of the diagnostic tools used during the clinical study. The opinions expressed in the manuscript are the author’s which do not necessarily reflect company policies. KA and DN have no conflicts to declare.

## Publisher’s Note

All claims expressed in this article are solely those of the authors and do not necessarily represent those of their affiliated organizations, or those of the publisher, the editors and the reviewers. Any product that may be evaluated in this article, or claim that may be made by its manufacturer, is not guaranteed or endorsed by the publisher.

## Supplementary Material

Supplementary Figure 1 Bacterial quantification of the TZP1 experiment. Box and whiskers plots show the fold change of the 16S rRNA gene count as generated by qPCR from DNA extracted from stool for all mice at each time point as compared to Day 8, the timepoint immediately prior to antibiotic treatment.

Supplementary Figure 2 PCoA of taxonomic profiles and immune status. PCoA analysis based on the Bray-Curtis distance matrix of OTUs from TZP2. Each circle represents a single mouse at a single timepoint. Closed circles represent immunocompetent mice and open circles represent neutropenic mice.

Supplementary Figure 3 **(A)** Phyla profiles. The relative abundance of the phyla is represented by stacked bars for each mouse and each day. **(A)** Portrays TZP1 in which TZP was administered days 7–13, **(B,C)** portray immunocompetent mice (mice 1–10) and neutropenic mice (mice 11–20), respectively, from TZP2 in which TZP was administered on days 15–18. **(B,C)** Phyla profiles. The relative abundance of the phyla is represented by stacked bars for each mouse and each day. **(A)** Portrays TZP1 in which TZP was administered days 7–13, **(B,C)** portray immunocompetent mice (mice 1–10) and neutropenic mice (mice 11–20), respectively, from TZP2 in which TZP was administered on days 15–18.

Supplementary Figure 4 **(A)** Genera profiles. The relative abundance of genera is represented by stacked bars for each mouse and each day. The top 25 genera are colored individually and all other genera are assigned to the “Other Taxa” group (light gray). **(A)** Portrays TZP1 in which TZP was administered days 7–13, **(B,C)** portray immunocompetent mice (mice 1–10) and neutropenic mice (mice 11–20), respectively, from TZP2 in which TZP was administered on days 15–18. **(B,C)** Genera profiles. The relative abundance of genera is represented by stacked bars for each mouse and each day. The top 25 genera are colored individually and all other genera are assigned to the “Other Taxa” group (light gray). **(A)** Portrays TZP1 in which TZP was administered days 7–13, **(B,C)** portray immunocompetent mice (mice 1–10) and neutropenic mice (mice 11–20), respectively, from TZP2 in which TZP was administered on days 15–18.

Supplementary Figure 5 **(A)** Potentially Resistant OTUs. OTUs that underwent a positive fold change of relative abundance post-TZP exposure are shown for each experiment (**A,B**, TZP1 and TZP2, respectively). Fold change was calculated compared to the last pre-antibiotic time point (Days 6 and 14, respectively). The vertical line indicates the approximate first TZP exposure. **(B,C)** Potentially resistant OTUs. OTUs that underwent a positive fold change of relative abundance post-TZP exposure are shown for each experiment (**A**,**B**, TZP1 and TZP2, respectively). Fold change was calculated compared to the last pre-antibiotic time point (Days 6 and 14, respectively). The vertical line indicates the approximate first TZP exposure.

Supplementary Figure 6 Piperacillin concentrations in mouse feces during and post-TZP exposure.

Supplementary Figure 7 Piperacillin concentrations in mouse feces during and post-TZP exposure.
