# Supplementary material for: Murine Model for Measuring Effects of Humanized-Dosing of Antibiotics on the Gut Microbiome
Source: Front Microbiol. 2022 Feb 17;13:813849. doi: 10.3389/fmicb.2022.813849 (PMC8892246; doi:10.3389/fmicb.2022.813849)
Supplement: Supplementary Figure 1 — Bacterial quantification of the TZP1 experiment. Box and whiskers plots show the fold change of the 16S rRNA gene count as generated by qPCR from DNA extracted from stool for all mice at each time point as compared to Day 8, the timepoint immediately prior to antibiotic treatment. [file Presentation_1.ppt]

## Slide 1
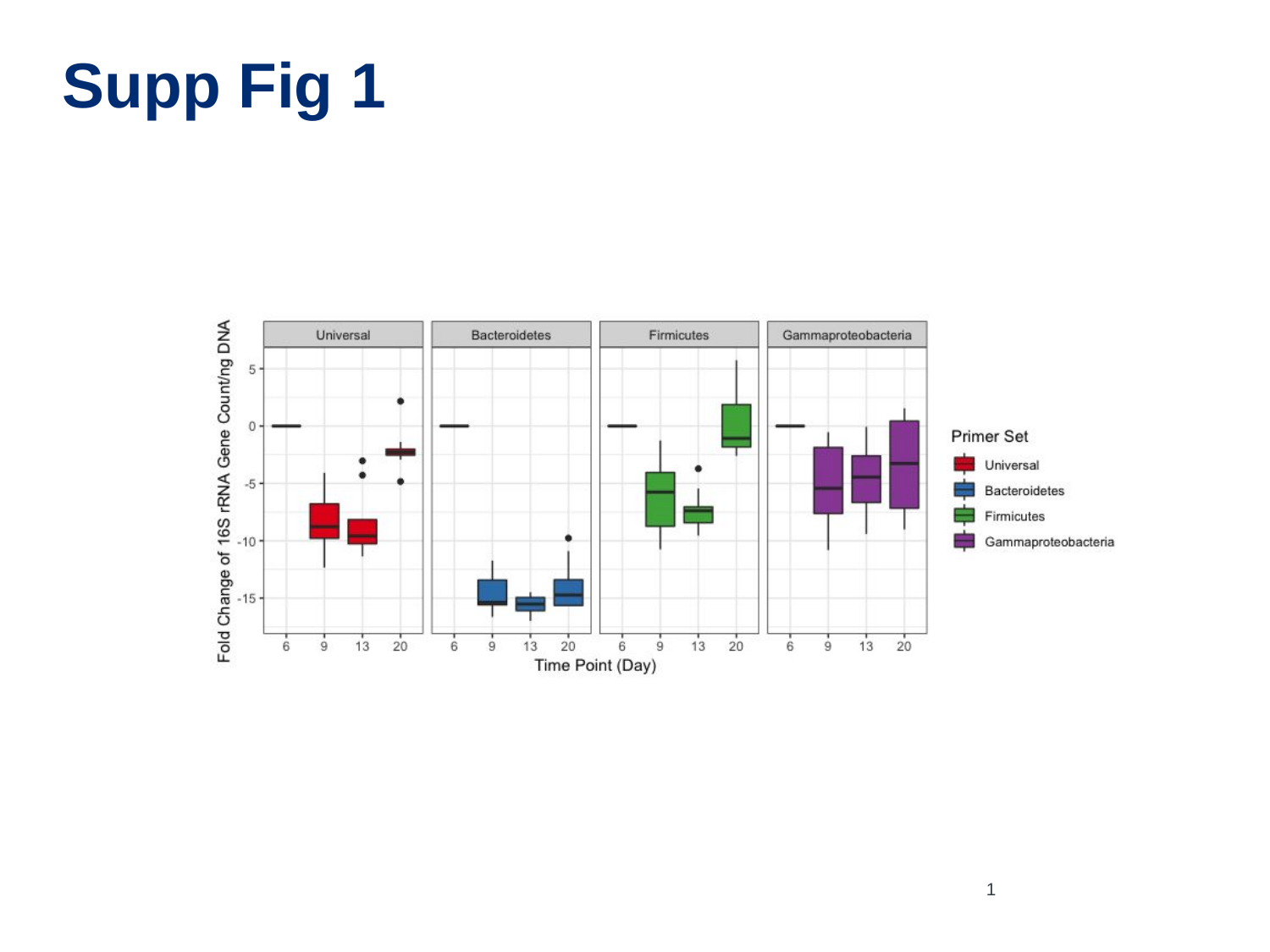

# Supp Fig 1
<number>

## Slide 2
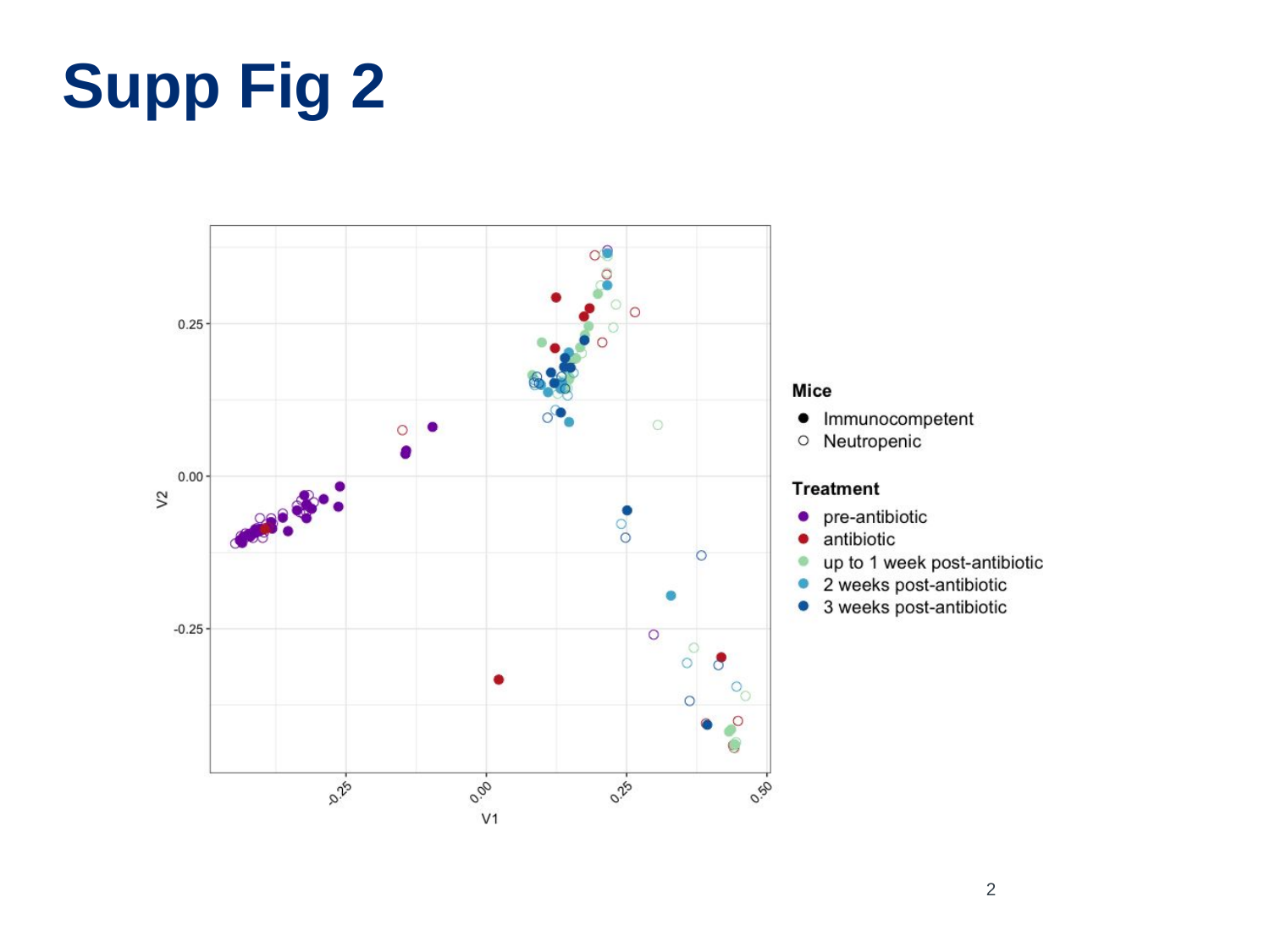

# Supp Fig 2
<number>

## Slide 3
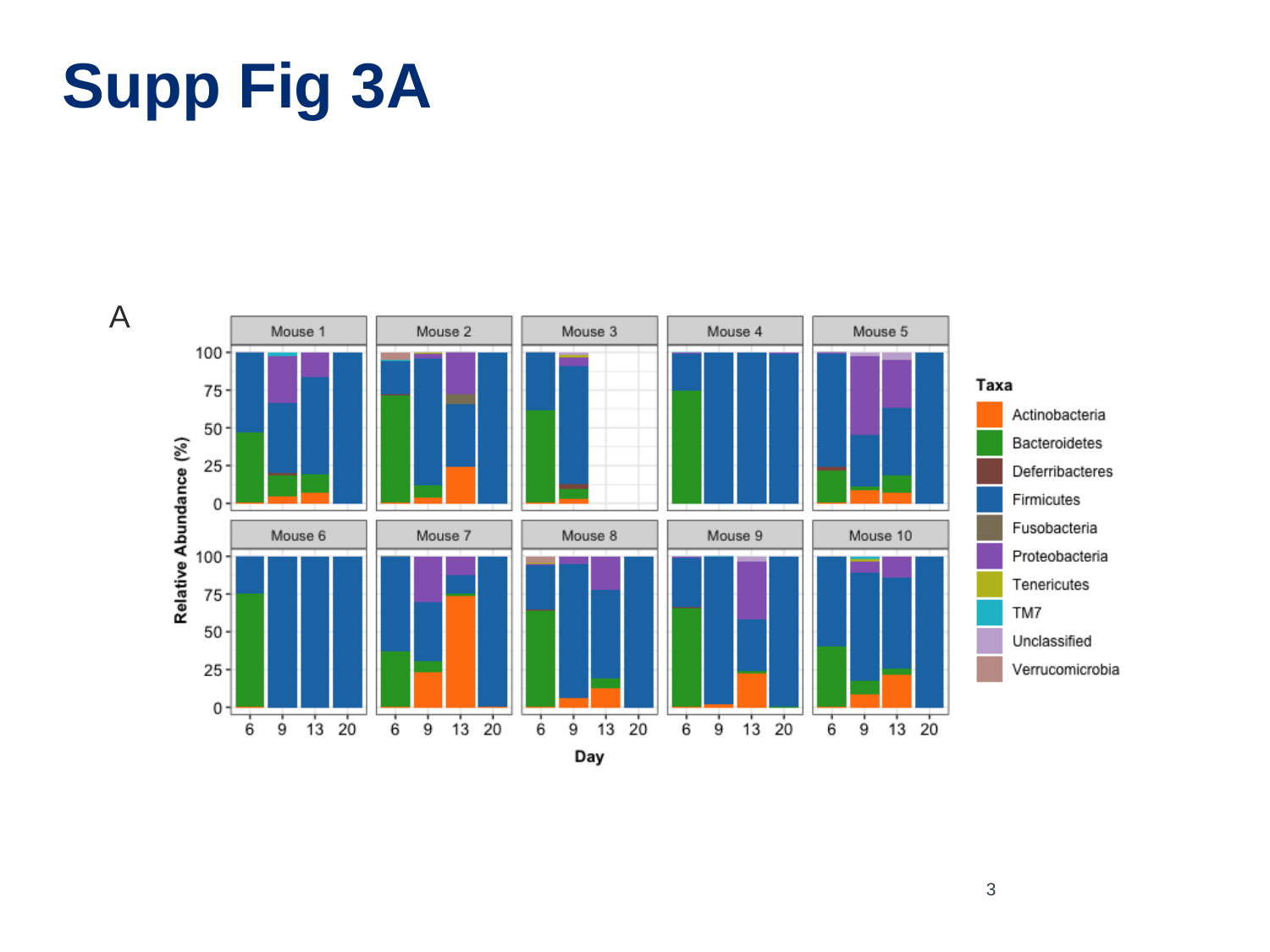

# Supp Fig 3A
A
<number>

## Slide 4
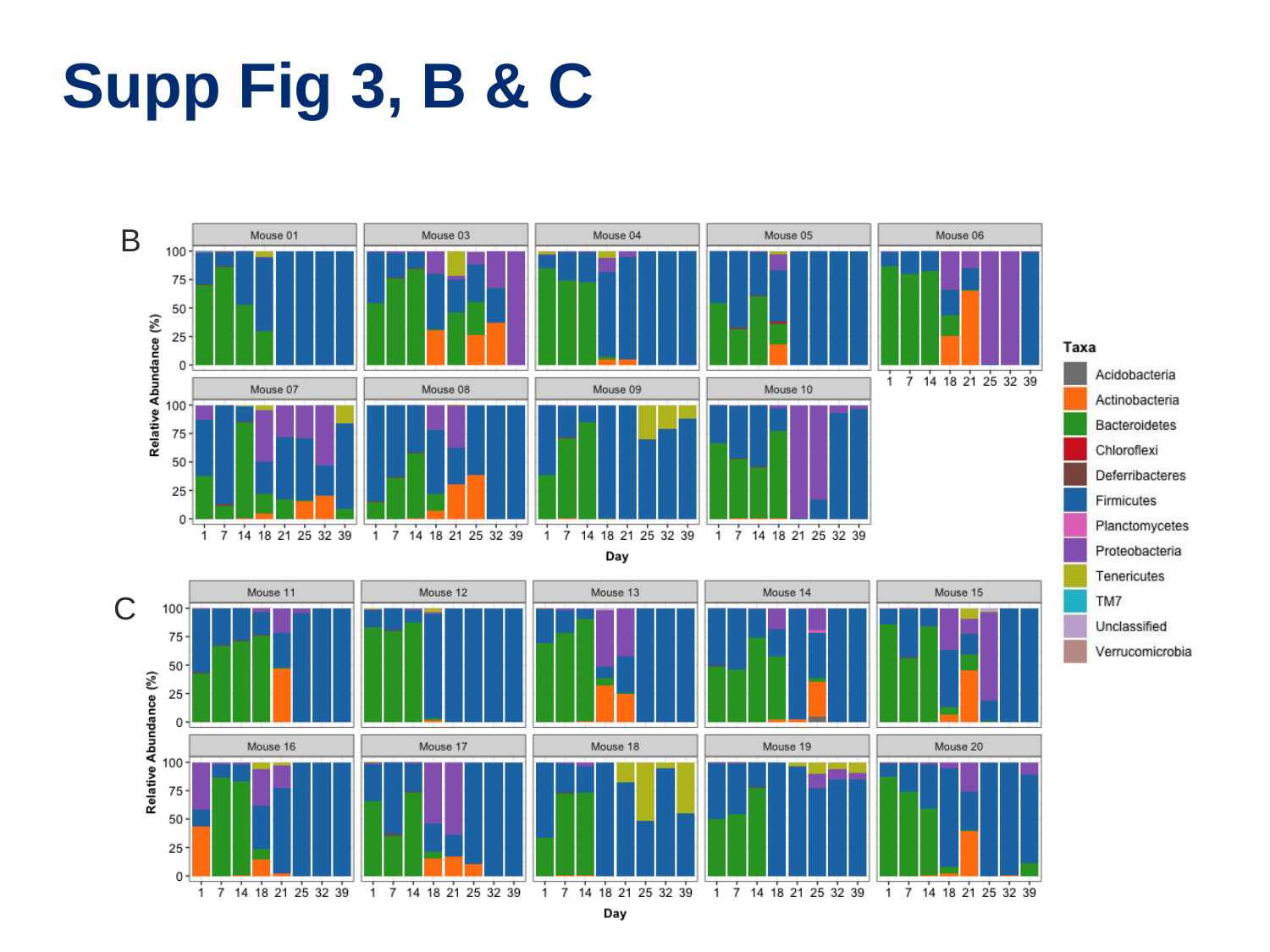

# Supp Fig 3, B & C
B
C
<number>

## Slide 5
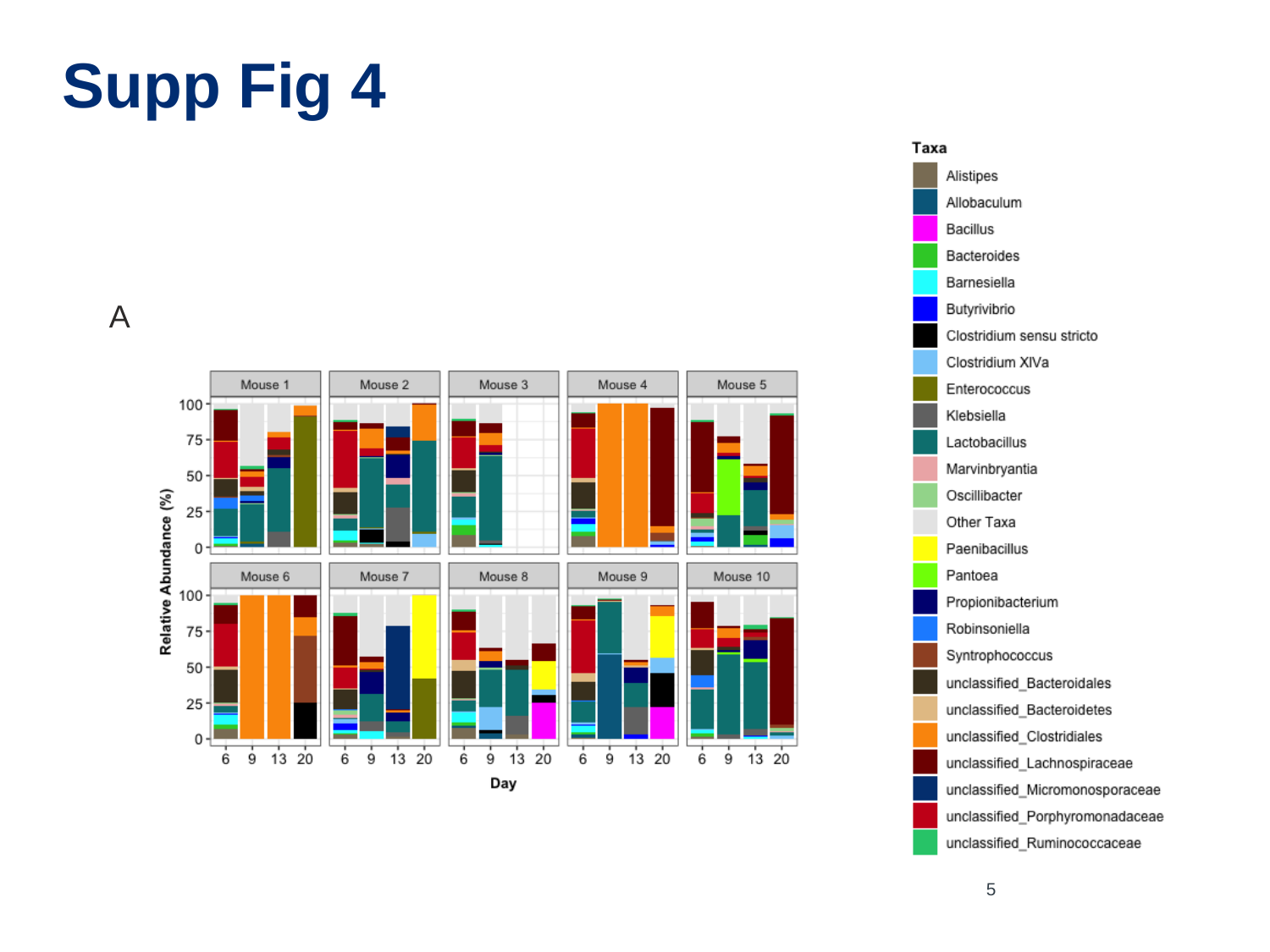

# Supp Fig 4
A
<number>

## Slide 6
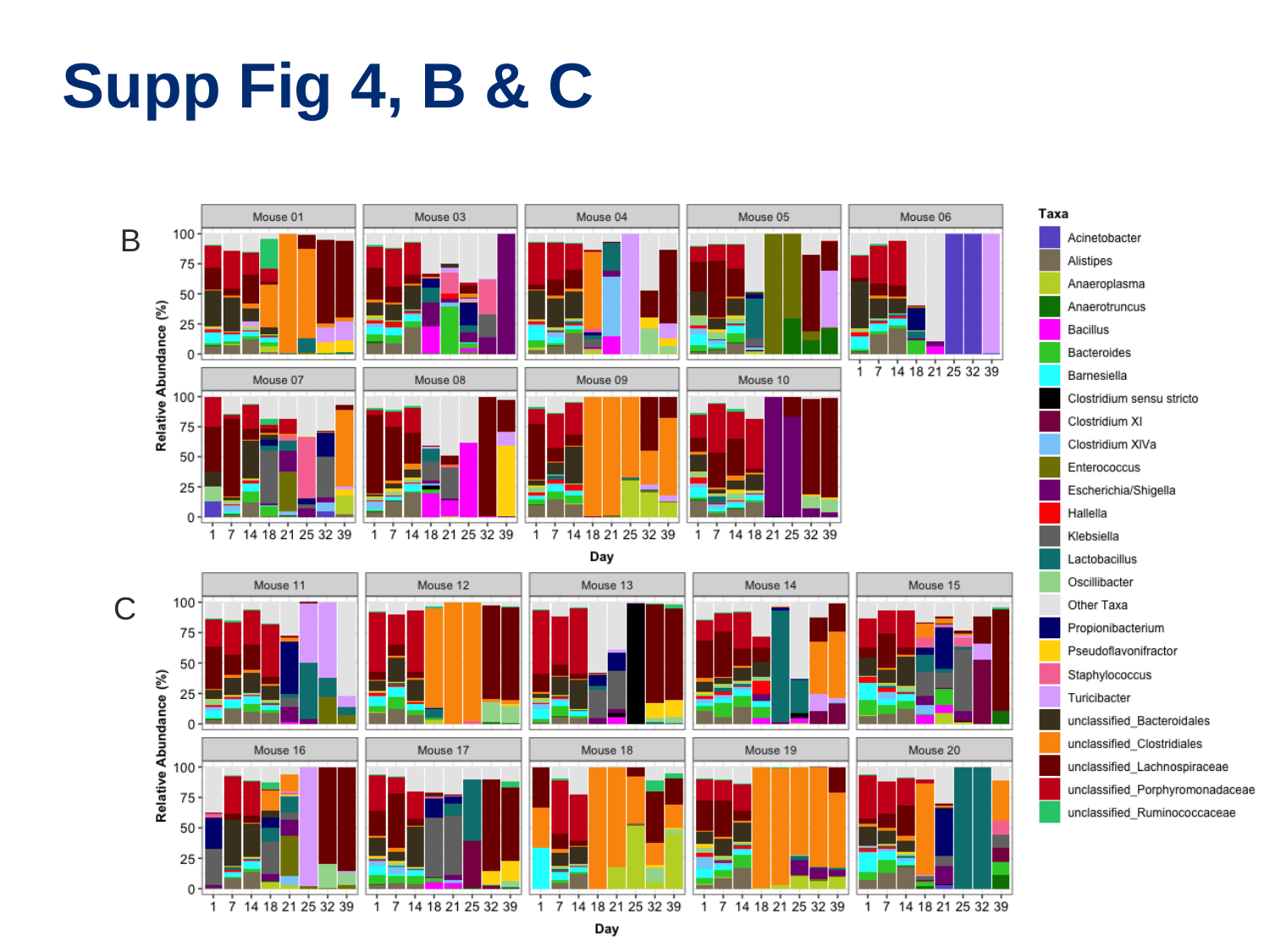

# Supp Fig 4, B & C
B
C
<number>

## Slide 7
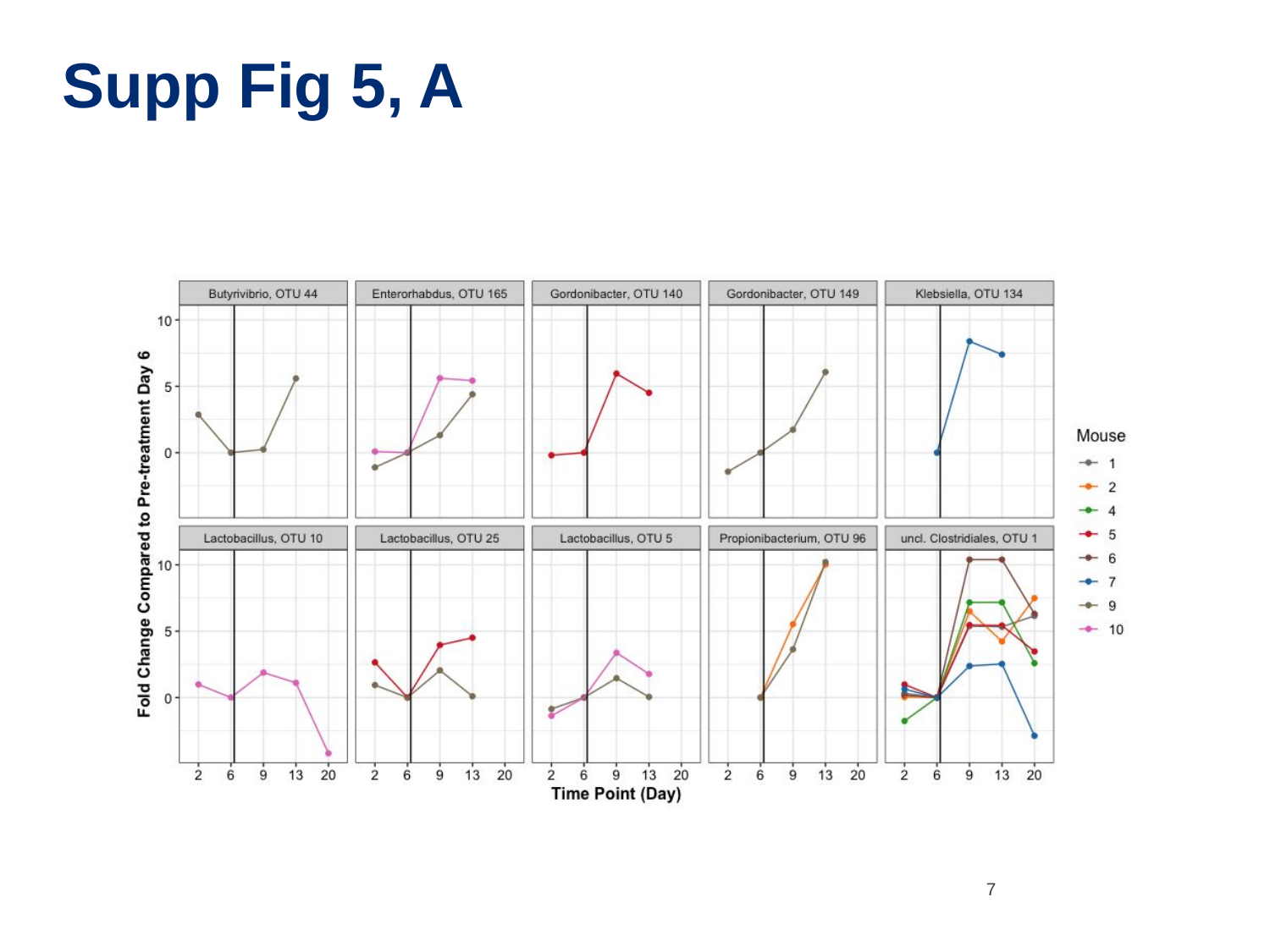

# Supp Fig 5, A
<number>

## Slide 8
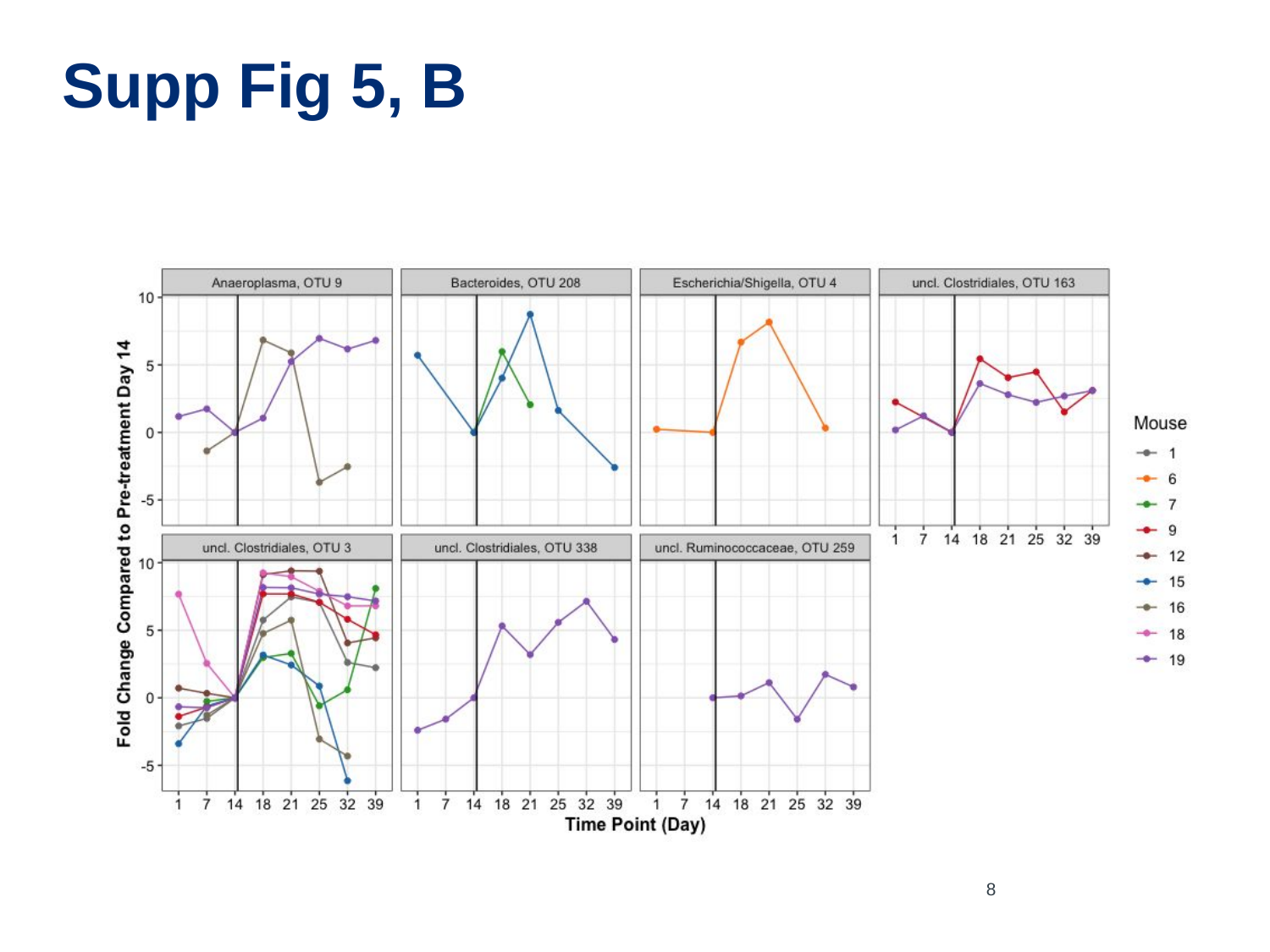

# Supp Fig 5, B
<number>

## Slide 9
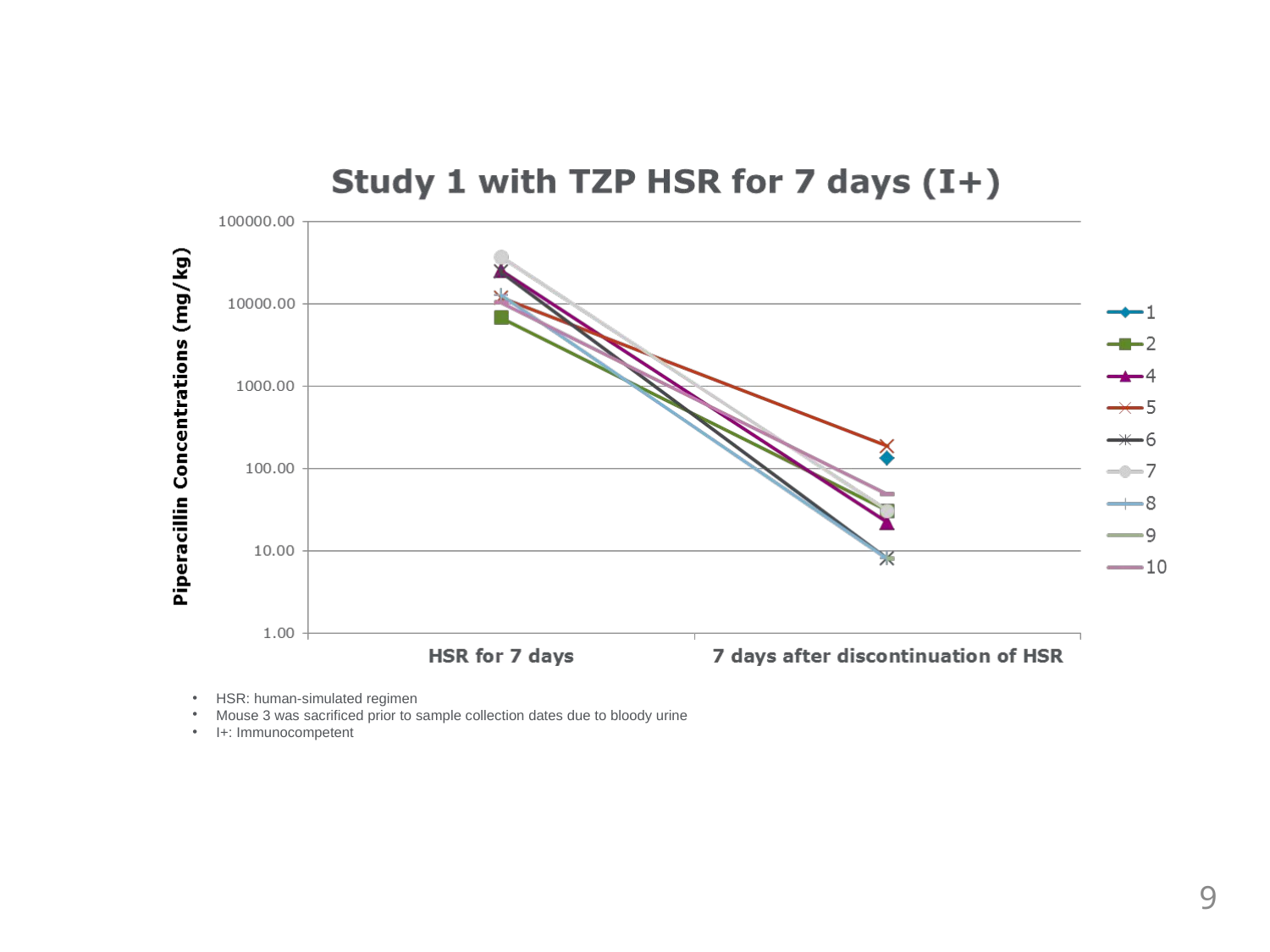

HSR: human-simulated regimen
Mouse 3 was sacrificed prior to sample collection dates due to bloody urine
I+: Immunocompetent
<number>

## Slide 10
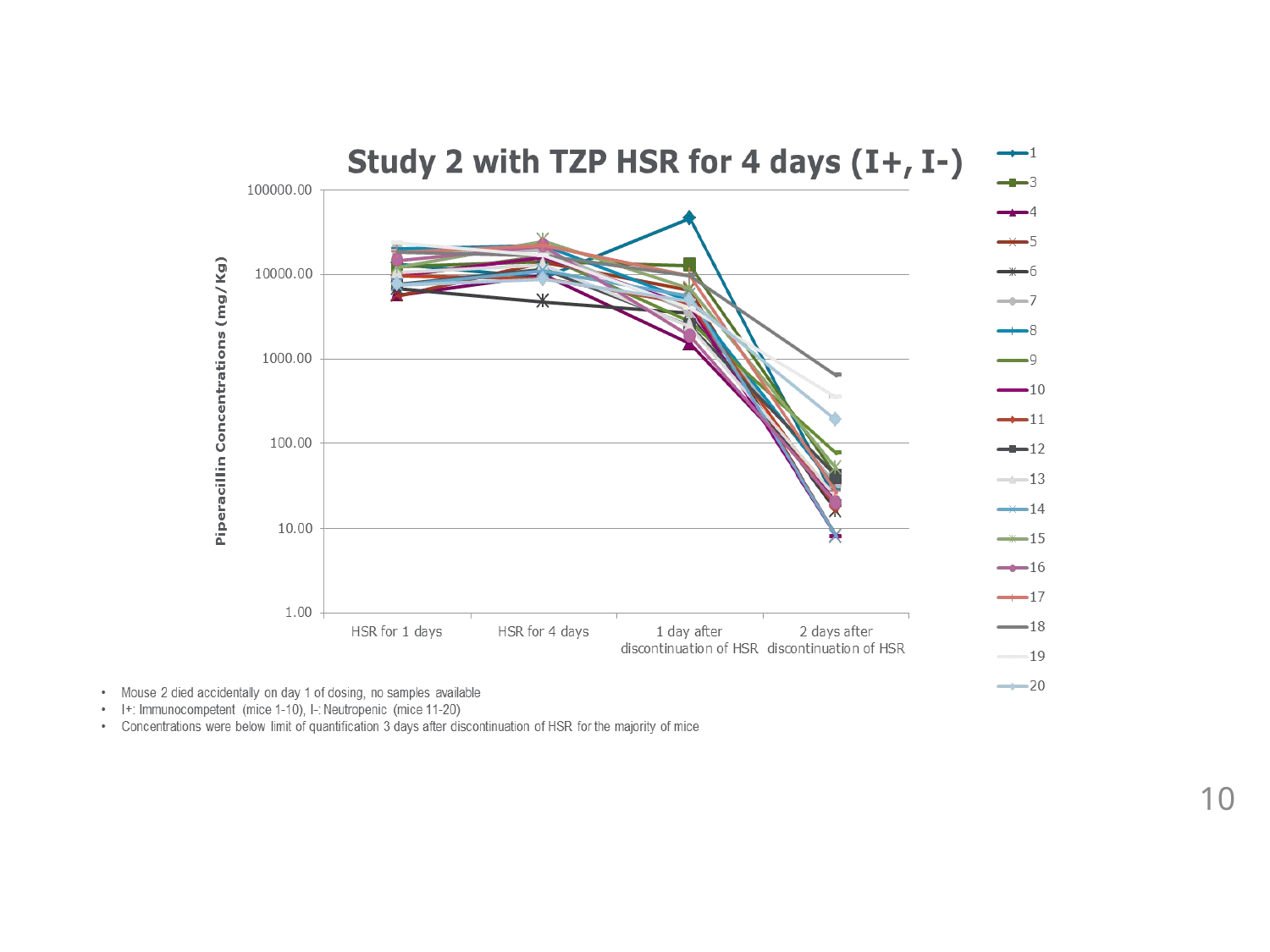

<number>
